# The SWI/SNF complex member SMARCB1 supports lineage fidelity in kidney cancer

**DOI:** 10.1016/j.isci.2023.107360

**Published:** 2023-07-13

**Authors:** Ludovic Wesolowski, Jianfeng Ge, Leticia Castillon, Debora Sesia, Anna Dyas, Shoko Hirosue, Veronica Caraffini, Anne Y. Warren, Paulo Rodrigues, Giovanni Ciriello, Saroor A. Patel, Sakari Vanharanta

**Affiliations:** 1 MRC Cancer Unit, University of Cambridge, Hutchison/MRC Research Centre, Cambridge Biomedical Campus, Box 197, Cambridge CB2 0XZ, UK; 2 Early Cancer Institute, Department of Oncology, University of Cambridge, Cambridge CB2 0XZ, UK; 3 Translational Cancer Medicine Program, Faculty of Medicine, Biomedicum Helsinki, University of Helsinki, 00014 Helsinki, Finland; 4 Department of Computational Biology, University of Lausanne (UNIL), 1015 Lausanne, Switzerland; 5 Swiss Cancer Center Leman, Lausanne, Switzerland; 6 Department of Histopathology, Cambridge University Hospitals NHS Foundation Trust, Cambridge CB2 0QQ, UK; 7 Department of Biochemistry and Developmental Biology, Faculty of Medicine, University of Helsinki, 00014 Helsinki, Finland

**Keywords:** Human genetics, Cellular physiology, Medical microbiology, Cancer

## Abstract

Lineage switching can induce therapy resistance in cancer. Yet, how lineage fidelity is maintained and how it can be lost remain poorly understood. Here, we have used CRISPR-Cas9-based genetic screening to demonstrate that loss of SMARCB1, a member of the SWI/SNF chromatin remodeling complex, can confer an advantage to clear cell renal cell carcinoma (ccRCC) cells upon inhibition of the renal lineage factor PAX8. Lineage factor inhibition-resistant ccRCC cells formed tumors with morphological features, but not molecular markers, of neuroendocrine differentiation. SMARCB1 inactivation led to large-scale loss of kidney-specific epigenetic programs and restoration of proliferative capacity through the adoption of new dependencies on factors that represent rare essential genes across different cancers. We further developed an analytical approach to systematically characterize lineage fidelity using large-scale CRISPR-Cas9 data. An understanding of the rules that govern lineage switching could aid the development of more durable lineage factor-targeted and other cancer therapies.

## Highlights

• A CRISPR screen identifies SMARCB1 as a lineage fidelity factor in RCC
• SMARCB1 loss can facilitate independence from the lineage factor PAX8 in RCC
• Morphological neuroendocrine features associated with PAX8 independence in RCC xenografts
• Large CRISPR screen datasets indicate widespread lineage factor independence in cancer

## Graphical abstract

**Figure:**
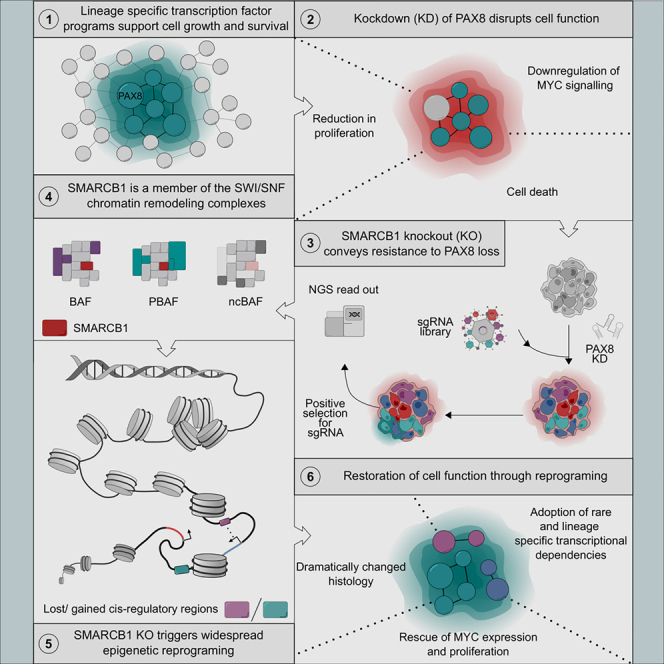

## Introduction

Lineage-specific transcription factors (TFs), such as SOX10 and MITF in melanoma, have emerged as a common class of essential genes in large-scale functional cancer cell line fitness screens.^1^^,^^2^ Clinically relevant examples include estrogen and androgen receptors, which are well-established therapeutic targets in breast and prostate cancer, respectively.^3^ The success of hormone therapies suggests that lineage factor dependencies could be exploitable for clinical benefit also in non-hormone receptor-driven cancers. However, the cancer-relevant biology of lineage factors and the mechanisms that maintain lineage fidelity, i.e., the dependency of a cancer on the transcriptional lineage factor programs of its tissue of origin, in advanced cancer clones remain poorly understood. It is also unclear what the long-term consequences of lineage factor inhibition are, how lineage factor independence may arise, and how lineage switching, or loss of lineage fidelity, an emerging mechanism of therapy resistance,^4^ is facilitated.

Clear cell renal cell carcinoma (ccRCC) is the most common form of kidney cancer with ∼300,000 diagnoses and ∼100,000 deaths annually worldwide.^5^ Inactivation of the von Hippel-Lindau tumor suppressor gene (*VHL*), seen in ∼90% of ccRCCs, is the only clonal genetic driver alteration in most ccRCCs.^6^ VHL loss leads to stabilization of the hypoxia-inducible factors HIF1A and HIF2A, of which HIF2A is critical for ccRCC development.^7^ Interestingly, HIF2A inhibitors have demonstrated efficacy against ccRCC in some patients, but *de novo* and acquired resistance are common.^8^^,^^9^ The widespread and uniform expression of PAX8,^10^ a well-established example of a lineage-specific transcription factor dependency,^1^^,^^2^^,^^11^^,^^12^^,^^13^^,^^14^ make PAX8 an attractive alternative target for ccRCC therapy, especially given the redundancy between Pax8 and Pax2 in normal renal development in mice.^12^ Recent evidence suggests that in ccRCC cells PAX8 maintains the expression of *CCND1* and *MYC*, two canonical oncogenes that are required for ccRCC proliferation.^14^ PAX8 regulates *CCND1* expression through a distal enhancer, the activity of which also depends on HIF2A, whereas PAX8-dependent *MYC* expression involves the downstream mediator HNF1B, another renal lineage factor.^14^

While ligand-independent transcription factors lack active pockets where small-molecule inhibitors could bind, emerging modalities, such as proteolysis-targeting chimeras or protein-protein interaction inhibitors, could expand the clinically druggable target space to include a broader set of transcription factors in the future.^15^^,^^16^ Meanwhile, detailed functional genetic modeling in experimental systems could help evaluate the potential of lineage factors as therapeutic targets. To understand the consequences of lineage factor inhibition, we have used genetic screening to identify mechanisms that maintain lineage fidelity and *PAX8* dependency in ccRCC cells. We find that loss of the SWI/SNF complex member SMARCB1 can facilitate the development of resistance to PAX8 inhibition, giving rise to a strongly altered histological appearance which displays morphological features of neuroendocrine differentiation but no molecular neuroendocrine markers. The dedifferentiated phenotype relies on newly acquired dependencies on *IRF2*, *BHLHE40*, and *ZNFX1*, which represent rare pan-cancer dependencies. Moreover, systematic analysis of hundreds of cell lines revealed evidence of molecular mechanisms that may promote lineage factor inhibition resistance across several different cancer lineages. We conclude that resistance to lineage factor inhibition follows molecular logic that could be exploited for the prevention of lineage switching, potentially leading to more sustained therapy responses to various anticancer agents.

## Results

### SMARCB1 loss facilitates resistance to lineage factor inhibition in ccRCC cells

**Figure 1:**
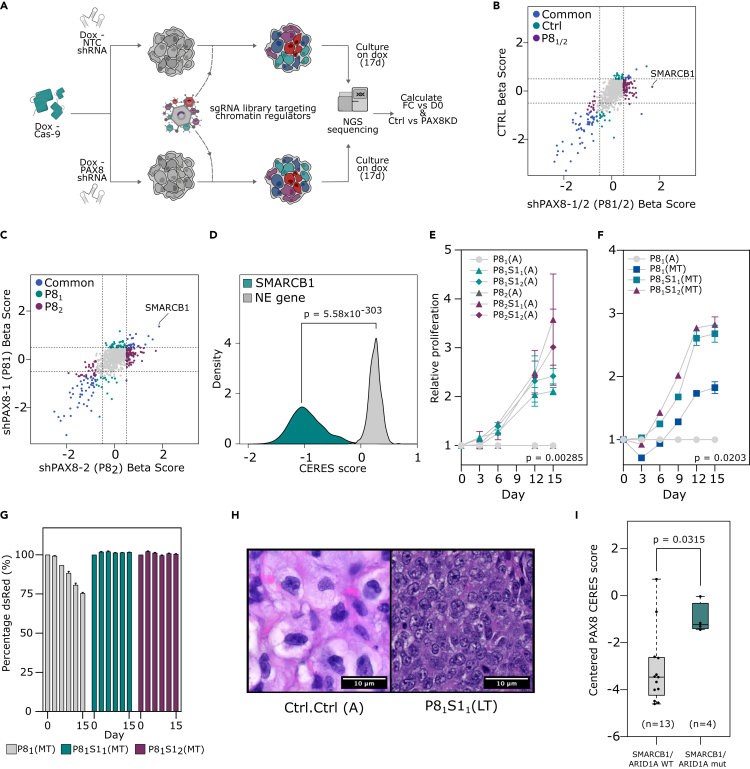
*SMARCB1* loss facilitates resistance to lineage factor inhibition in ccRCC cells (A) A schematic of a pooled CRISPR-Cas9-based loss-of-function screen using an sgRNA library targeting chromatin regulators to identify genes capable of modulating the dependency of ccRCC cell lines on PAX8 inhibition. (B and C) Changes in sgRNA abundance over time, measured by calculating beta scores using the top three enriched sgRNAs per gene relative to day 0, from two technical replicates. (B) The beta scores for the control arm of the screen versus the pooled experimental arm (P8_1/2_). (C) The beta scores for each of the experimental arms of the screen. Highlighted points have a beta score <-0.5 or >0.5 and a p value <0.05. p-value was calculated by permutation based approach using MAGeCK. (D) Genetic dependency data for 946 cell lines from the DepMap project. Distribution of CERES scores for the gene *SMARCB1* and an example non-essential gene (*CNBD1*). Kruskal-Wallis test. (E and F) Competitive proliferation assays using 786-M1A Cas9 clone 6 (786-M1A-C6) cells expressing combinations of sgRNAs (Ctrl or S1_1/2_) and inducible shRNAs (Ctrl or P8_1/2_), competed against cells with a *PAX8* KD (P8_1/2_). Three technical replicates per condition. (E) Doxycycline was added at day 0 (acute, A), (F) cells were pre-treated for ∼1 month before the start of the assay (midterm, MT). Error bars are SD. Kruskal-Wallis test. (G) Escaper assay with three technical replicates using 786-M1A-C6 cells, showing the percentage of cells expressing the fluorophore dsRed as a measure of PAX8 shRNA expression for cells pre-cultured on doxycycline for ∼1 month (MT), normalized to day 0. Cells were sorted at the beginning of the assay to ensure a starting point of 100% dsRed. Error bars are SD. (H) Hematoxylin and eosin (H&E) staining of tumors formed by Ctrl.Ctrl and P8_1_S1_1_(LT) cells, respectively. (I) Boxplot of *PAX8*-centered CERES dependency score for *VHL*-mutant RCC lines from the DepMap project, which are either WT (n = 13) or mutant for *ARID1A* (n = 1) or *SMARCB1* (n = 3). Kruskal-Wallis test. For boxplots, center line shows the median, the box bounds represent the first and third quartiles, and the whiskers extend to the highest and lowest values. See also Figures S1 and S2.

To identify genes that maintain the dependency of ccRCC cells on PAX8, we performed loss-of-function screening on a PAX8 knockdown (KD) background in 786-M1A cells, a metastatic *VHL* mutant ccRCC cell line,^17^ using a single guide RNA (sgRNA) library targeting chromatin regulators,^18^ key factors involved in the maintenance of cellular identity (Figure 1A). To establish the optimal conditions for screening, we derived a doxycycline-inducible Cas9 clone with high editing efficiency and validated that two short hairpin RNAs (shRNAs) targeting *PAX8* could effectively suppress PAX8 expression within 24 h and induce a negative proliferative phenotype (Figures S1A–S1E). The shRNAs were co-expressed with a fluorescent reporter, which allowed us to monitor expression and select for cells that maintained *PAX8* KD throughout the experiment (Figure S1F). All genes and 98.5% of sgRNA were captured at the start of the screen and there was a negative selection against known essential genes but not non-essential genes (Figures S1G–S1I). The most enriched *PAX8* KD condition (P8_1/2_)-specific hit from the screen was SMARCB1, a member of the SWI/SNF chromatin remodeling complex, which has a key role in chromatin landscape maintenance in development and tissue homeostasis (Figures 1B and 1C).^19^ Three additional SWI/SNF complex members were also specifically enriched in the *PAX8* KD condition, of which ARID1A showed the strongest enrichment (Figure S1J). ARID1A and SMARCB1 inactivation have recently been shown to reduce the sensitivity of breast cancer cells to estrogen receptor inhibition,^20^^,^^21^ giving support to our findings in a second cancer type. Interestingly, unlike *ARID1A*, and in contrast to its effect in our screen, *SMARCB1* is a prototypical pan-cancer essential gene (Figures 1D, S1K, and S1L).

We validated the results from the screen by competitive cellular proliferation assays using two additional SMARCB1 sgRNAs (S1_1/2_) (Figures 1E and S1M). Acute *PAX8* KD/*SMARCB1* knockout (KO) (P8_1/2_S1_1/2_(A)) resulted in a proliferative rescue but the cells grew less stably and were sensitive to passaging. Given time (∼1 month, mid-term, MT), they began to grow more robustly (Figures 1F and S2A). *SMARCB1* loss thus provides an immediate proliferative advantage to *PAX8* KD cells but with a significant stability trade-off that can be selected against over time. Over a period of ∼2–3 months (long-term, LT), the P8_1_S1_1/2_(LT) cells maintained a similar growth phenotype but cells without *SMARCB1* KO (P8_1_Ctrl) also adapted to PAX8 suppression (Figures S2B and S2C). Critically, despite a partial proliferative rescue, a selective pressure to regain PAX8 suppression was maintained in P8_1_Ctrl(LT/MT) cells but not in their *SMARCB1* KO counterparts, as evidenced by the gradual loss of PAX8 shRNA expression in P8_1_Ctrl(LT/MT) cells (Figures 1G and S2D). Tumor growth *in vivo* was totally abrogated by *PAX8* KD (P8_1_Ctrl(A)), partially rescued for P8_1_Ctrl(LT) cells and completely rescued for P8_1_S1_1_(LT) cells, which were transduced with the more efficient *SMARCB1* sgRNA (Figures S1M, S2A, and S2H). P8_1_S1_2_(LT) cells, on the other hand, showed only weak tumorigenicity (Figure S2E). Histological analysis revealed a high-grade ccRCC phenotype with sarcomatoid dedifferentiation in the control cells, characteristic of the 786-M1A cells.^17^ On the other hand, tumors arising from the *PAX8* KD background presented as high-grade undifferentiated carcinomas, with extensive areas of necrosis and morphological appearance reminiscent of neuroendocrine differentiation, in keeping with a large-cell phenotype, but no morphological evidence of rhabdoid dedifferentiation (Figures 1H and S2F). In agreement with the *in vitro* data, P8_1_Ctrl(LT) tumors displayed also areas of the original histology, possibly indicating the presence of escapers from *PAX8* KD. However, immunohistochemical and RNA analyses did not detect expression of typical neuroendocrine markers specifically associated with the *PAX8* KD-resistant phenotype (Figure S2G). Interestingly, a similar phenotype, morphological neuroendocrine features without molecular neuroendocrine markers, has recently been described in an experimental mouse-derived renal carcinoma model that displays molecular features of an aggressive ccRCC subtype.^22^

To expand our study to additional ccRCC models, we took a systematic approach and identified *VHL* mutant ccRCC cell models which have a non-synonymous and predicted damaging or TCGA/COSMIC hotspot *SMARCB1* (n = 3) or *ARID1A* (n = 1) mutation from the cell line encyclopedia (CCLE)^23^ and compared their PAX8 inhibition sensitivity to their *SMARCB1*/*ARID1A* wild-type (WT) counterparts (n = 13) using loss-of-function data from the cancer dependency map project (DepMap).^24^ In line with the findings from our screen, *SMARCB1*/*ARID1A* mutant lines showed strong resistance to *PAX8* KO compared to their WT counterparts (Figure 1I). However, there were also two *VHL* mutant cell lines that showed similar resistance to *PAX8* KO but did not have a *SMARCB1* or *ARID1A* mutation, indicating that PAX8 inhibition resistance can arise through several mechanisms. In line with this, PAX8 depletion in UOK101 cells, another *VHL* mutant ccRCC cell line, resulted in quick emergence of a resistant population which maintained the essential status of *SMARCB1* (Figures S2H and S2I). In sum, inactivation of *SMARCB1*, a generally essential gene in cancer cells, is associated with lineage factor independence in ccRCC, but other mechanisms of lineage factor independence also exist.

### Large-scale alterations in enhancer activation states upon SMARCB1 loss

**Figure 2:**
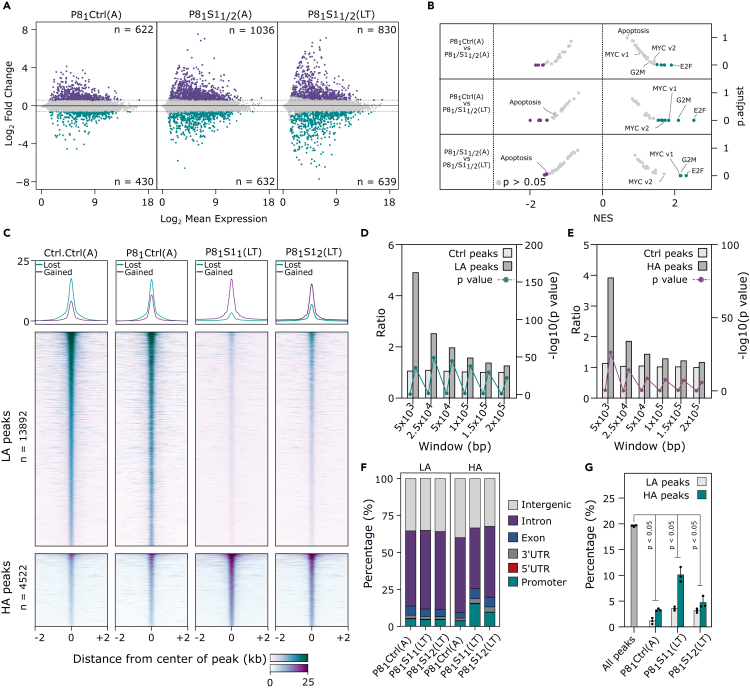
Large-scale alterations in enhancer activation states upon SMARCB1 loss (A) MA plots of RNA-seq differential expression analysis from 786-M1A-C6 cells. Gene expression fold change was calculated relative to shRen/sgNTC (Ctrl.Ctrl) cells. Highlighted points have an FC > 1.5 or <(-1.5) and p.adjust <0.05. Adjusted p values calculated with DEseq2. (B) Gene set enrichment analysis (GSEA) using the hallmarks collection from mSigDB, for different comparisons as indicated on the left. Highlighted points (purple/cyan) have a p.adjust <0.05. (C) Heatmaps showing normalized ATAC-seq signal +/− 2kb centered on summits of differentially accessible (DA) regions, defined by Ctr.Ctrl vs. P8_1_S1_1/2_ (FC > 2 or <(-2), p.adjust <0.001). Top panels show the average signal for higher accessible and lower accessible regions. (D and E) Correlation of ATAC-seq and transcriptional changes, for Ctrl.Ctrl(A) vs. P8_1_S1_1/2_(LT). Downregulated genes near lower accessibility regions in (D). Upregulated genes near higher accessibility regions in (E). Left y axis, the ratio of the number of downregulated/upregulated genes found within windows created around lower/higher accessible regions compared to the number of expressed genes (universe) also found within the windows. Right y axis, p value, one-tailed hypergeometric test. Matched Ctrl peaks for LA and HA regions were generated from the consensus list of all peaks merged across conditions. (F) Stacked bar plots of genomic annotations for LA and HA regions from comparisons Ctrl.Ctrl(A) vs. P8_1_Ctrl(A), P8_1_S1_1_(LT), and P8_1_S1_2_(LT) (FC > 2 or <(-2), p.adjust <0.001). (G) Percentage of regions annotated as a promoter in the consensus list of all peaks merged across conditions (dark gray) and lower and higher accessible regions from (F). Two-tailed hypergeometric test. See also Figure S3 and Tables S1, S2, S3, S4, S5, and S6.

To understand the role of SMARCB1 in PAX8 inhibition resistance, we performed RNA sequencing (RNA-seq) and assay for transposase-accessible chromatin using sequencing (ATAC-seq) to measure changes in the transcriptome and chromatin accessibility upon *SMARCB1* loss. We detected differentially expressed genes across conditions, with more gene expression changes in the P8_1_S1_1/2_(MT/LT) conditions compared to P8_1_Ctrl(A) (Figure 2A and Tables S1, S2, S3, S4, S5, and S6). In accordance with the proliferative phenotype, we found that *SMARCB1* loss triggered an increase in proliferative gene signatures (MYC_V1, MYC_V2, G2M, E2F), which increased over time (Figure 2B). The hallmark apoptosis signature was also reduced in the comparison between acute and long-term *SMARCB1* KO, supporting our observation that SMARCB1 simultaneously triggers heightened proliferation and instability, and over time clones which can tolerate *SMARCB1* loss are selected for (Figure 2B). From our ATAC-seq experiment, we detected ∼72,000 high confidence peaks in total across conditions, with control and P8_1_Ctrl(A) having a similar number of peaks and P8_1_S1_1/2_(LT) having substantially less (Figure S3A). There was a large proportion of differentially accessible regions in the *SMARCB1* KO conditions compared to the control, 18,414 in total, 13,892 of which had lower accessibility (LA) and 4,522 higher accessibility (HA) (Figures 2C, S3B, and S3C). P8_1_S1_1_(LT) and P8_1_S1_2_(LT) showed a similar overall pattern of altered DNA accessibility (Figure 2C), but the changes were more pronounced in P8_1_S1_1_(LT) cells (Figure S3B). As expected, increased chromatin accessibility was associated with increased gene expression whereas reduced chromatin accessibility was associated with reduced gene expression (Figures 2D, 2E, S3D, and S3E). We annotated the differentially accessible regions based on their location in the genome and found that the majority were intronic and intergenic and that there was a statistically significant underrepresentation of promoter annotations (Figures 2F and 2G). To test whether these regions were enhancers, we looked for an overlap with markers of active chromatin. A re-analysis of H3K27ac and H3K4me1 chromatin immunoprecipitation sequencing data in 786-M1A cells^25^ showed a typical bimodal distribution of average signal flanking the center of the ATAC-seq peaks for both LA and HA regions (Figures S3F and S3G). In summary, *SMARCB1* KO triggers large-scale enhancer re-programming in association with resistance to PAX8 suppression.

### Dedifferentiation in lineage factor inhibition-resistant ccRCC cells

**Figure 3:**
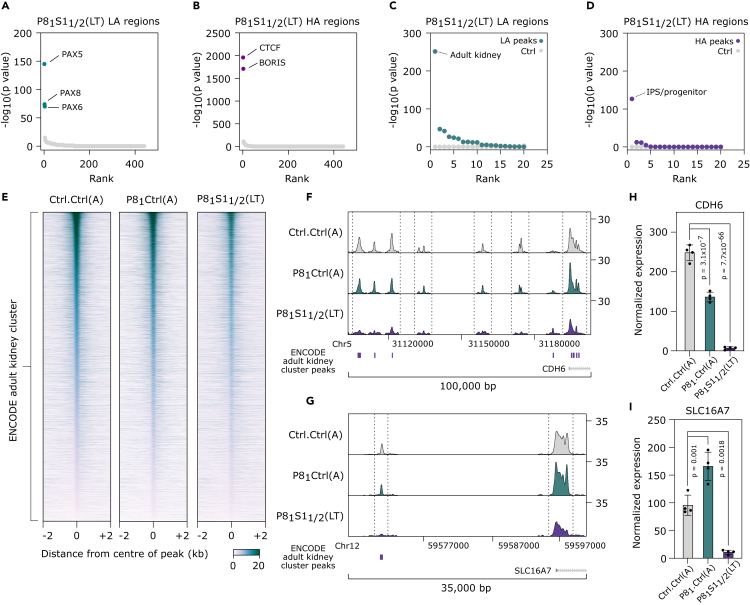
Loss of accessibility at lineage-specific gene regulatory elements in lineage factor inhibition-resistant ccRCC cells (A and B) Ranked plots of DNA motif analysis using a database of known motifs for the already defined (A) lower and (B) higher accessibility regions, from Ctrl.Ctrl(A) vs. P8_1_S1_1/2_(LT). p-values calculated using Homer. (C and D) Ranked plots of overlap analysis between cluster-specific peak sets generated from (Figure S5A) and lower (C) and higher (D) accessible region sets, from Ctrl.Ctrl(A) vs. P8_1_S1_1/2_(LT). One-tailed hypergeometric test. (E) Heatmaps showing normalized ATAC-seq signal +/− 2kb centered on peak summits for the ENCODE adult kidney cluster region set. (F and G) An example region of lost ATAC-seq signal at the *CDH6* (F) and the *SLC16A7* (G) loci. (H and I) Normalized mRNA expression of *CDH6* (H) and *SLC16A7* (I) as determined by RNAseq. Adjusted p values determined by DESeq2. Error bars are SD. See also Figures S4 and S5.

Enhancers are key mediators of lineage specification and the SWI/SNF complexes have been demonstrated to maintain tissue-specific enhancers,^26^^,^^27^ suggesting the possibility that lineage re-programming or dedifferentiation could underlie PAX8 inhibition resistance following *SMARCB1* KO. To test this hypothesis, we first performed motif analysis for our differentially accessible peak sets. As expected, the PAX motif was highly enriched in the LA set using both known and *de novo* motif analysis for P8_1_S1_1/2_(LT) and P8_1_Ctrl(A) (Figures 3A and S4A–S4D). Interestingly, the most specifically enriched motif for the P8_1_S1_1/2_ HA peak set was CTCF/BORIS, which has been linked to the formation of *SMARCB1* mutant rhabdoid tumors and the maintenance of a naive pluripotent stem cell state^28^^,^^29^ (Figures 3B and S4E–S4H).

To functionally annotate the differentially accessible enhancers in PAX8 inhibition-resistant cells, we downloaded 504 DNAase open chromatin profiles from the ENCODE project, spanning a range of adult and developmental cell types, and clustered the samples into tissue-specific clusters (Figure S5A). We derived sets of peaks that showed specificity for each cluster and ran an overlap analysis with our differentially accessible regions. The kidney-specific clusters were most enriched for the lower accessibility peaks sets for both P8_1_Ctrl and P8_1_S1_1/2_ (Figures 3C and S5B). However, the global loss of signal at these peak sets was substantially greater for P8_1_S1_1/2_ compared to P8_1_Ctrl, suggesting that *SMARCB1* loss triggers a widespread loss of renal epithelial epigenetic identity (Figure 3E). This was supported by specific genomic loci harboring known proximal tubule marker genes as defined by single-cell RNA-seq experiments, for example, *CDH6* and *SLC16A7*^30^ (Figures 3F–3I). The higher accessibility peaks for P8_1_S1_1/2_ overlapped most strongly with an IPS/progenitor cluster, which was not significantly enriched in the P8_1_Ctrl higher accessibility regions (Figures 3D and S5C–S5F).

**Figure 4:**
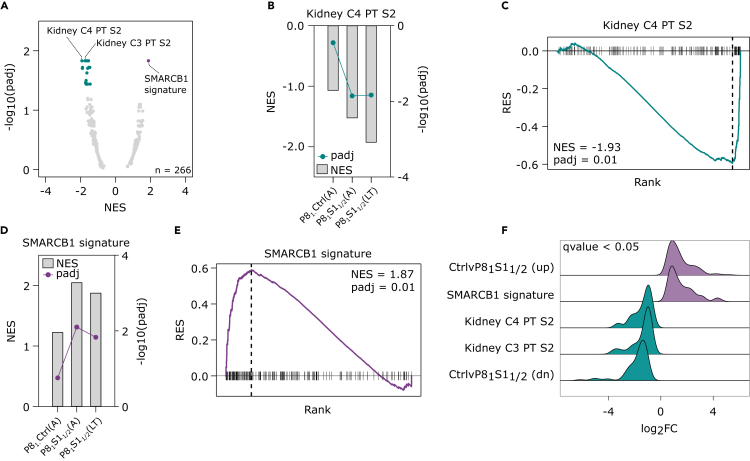
Dedifferentiation at the transcriptional level in lineage factor inhibition-resistant ccRCC cells (A) Volcano plot of GSEA with cell-type-specific transcriptional signatures from mSigDB collection 8, supplemented with a SMARCB1 signature (see STAR Methods), for the comparison Ctrl.Ctrl(A) vs. P8_1_S1_1/2_(LT). Highlighted points (purple/cyan) have a p.adjust <0.05. (B) Kidney proximal tubule C4 signature normalized enrichment scores (NES) from GSEA, for Ctrl.Ctrl(A) vs. P8_1_Ctrl(A), P8_1_S1_1/2_(A), and P8_1_S1_1/2_(LT). (C) GSEA plot of Kidney proximal tubule C4 signature for Ctrl.Ctrl(A) vs. P8_1_S1_1/2_(LT). (D) SMARCB1 signature NES from GSEA, for Ctrl.Ctrl(A) vs. P8_1_Ctrl(A), P8_1_S1_1/2_(A), and P8_1_S1_1/2_(LT). (E) GSEA plot of SMARCB1 signature for Ctrl.Ctrl(A) vs. P8_1_S1_1/2_(LT). (F) Ridge plot of GSEA result from the comparison of ccRCC CCLE lines, *SMARCB1* wild type vs. mutant from Figure 1I, using the SMARCB1, proximal tubule C3/C4 and upregulated and downregulated genes from Ctrl.Ctrl(A) vs. P8_1_S1_1/2_(LT). See STAR Methods for signature generation.

The global loss of the renal epithelial signal in conjunction with the gain of IPS/progenitor features at the chromatin accessibility level supports the notion that SMARCB1 may maintain a lineage-differentiated cellular state, the loss of which promotes PAX8 inhibition resistance. To test this at the level of gene expression, we used the mSigDB cell-type-specific signature collection (C8), supplemented with a signature that we derived from *SMARCB1* re-introduction experiments in rhabdoid tumor cell lines.^31^ The two most significantly downregulated signatures in the *SMARCB1* KO lines were from renal proximal tubules, the proposed origin of ccRCC (Figure 4A). The loss of renal transcriptional identity followed a similar pattern to the chromatin accessibility changes: *PAX8* KD alone showed a negative enrichment for the proximal epithelial signature C4 but failed to reach significance (p < 0.05) and P8_1_S1_1/2_(A) showed significant downregulation of the signature which reduced further over time (P8_1_S1_1/2_(LT)) (Figures 4B and 4C). Similarly, *PAX8* KD alone induced a positive enrichment of the rhabdoid SMARCB1 signature, but significance was only reached when *SMARCB1* was also knocked out (Figures 4A, 4D, and 4E). Upregulated and downregulated signatures derived from our RNA-seq data were also significantly positively and negatively enriched, respectively, in the *SMARCB1* mutant ccRCC cell lines in the CCLE dataset, suggesting that a similar mechanism accounts for the PAX8 inhibition insensitivity in these models (Figure 4F). In summary, PAX8 inhibition-resistant ccRCC cells display a global reduction in the kidney-specific *cis*-regulatory and transcriptional programs in favor of a dedifferentiated state which shares molecular features of *SMARCB1* loss in pediatric rhabdoid tumors.

### Acquired requirement of rare transcriptional dependencies in lineage factor inhibition-resistant ccRCC cells

**Figure 5:**
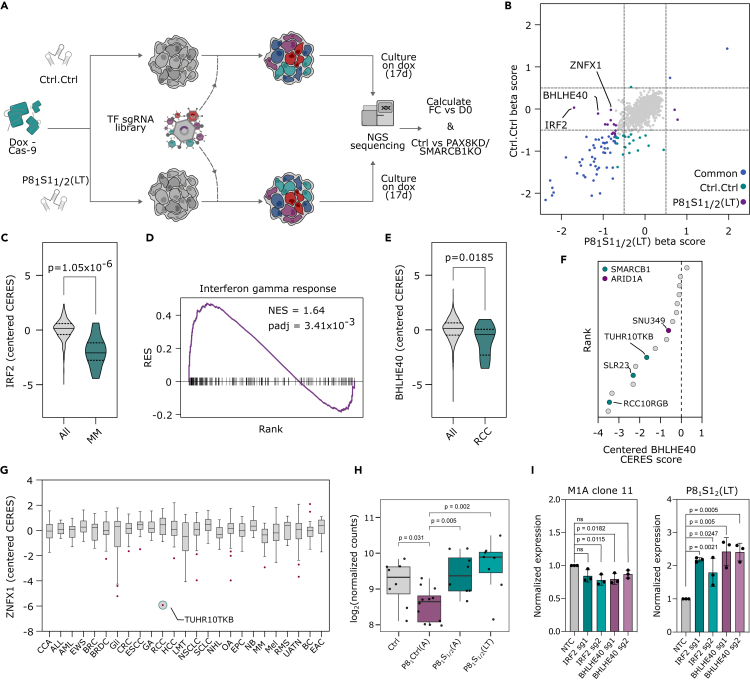
Acquired requirement of rare transcriptional dependencies in lineage factor inhibition-resistant ccRCC cells (A) Schematic overview of a CRISPR-Cas9 screen to look for new transcriptional dependencies in *PAX8* KD/*SMARCB1* KO cells. (B) The beta scores for the control arm (Ctrl.Ctrl) of the screen versus the pooled experimental arm (P8_1_S1_1/2_(LT)). Beta scores were calculated using the fold change of the top three depleted sgRNAs per gene relative to the plasmid library, from two replicates. p-value calculated by permutation method using MAGeCK. (C) Genetic dependency data from the DepMap project. *IRF2* centered CERES dependency score of multiple myeloma (MM) cell lines (n = 21) versus cells from all other lineages (n = 767). Kruskal-Wallis test. (D) GSEA plot of interferon-gamma response signature from mSigDB hallmarks collection for P8_1_Ctrl(A) vs. P8_1_S1_1/2_(LT). (E) Genetic dependency data from the DepMap project. *BHLHE40*-centered CERES dependency score for RCC cell lines (n = 23) versus cells from all other lineages (n = 765). Kruskal-Wallis test. (F) *VHL*-mutant ccRCC DepMap cell lines ranked by *BHLHE40*-centered CERES dependency score. *SMARCB1* and *ARID1A* mutant cell lines from Figure 1I. (G) Genetic dependency data from the DepMap project. *ZNFX1*-centered CERES dependency scores across 25 lineages, with ≥10 cell lines per lineage. CCA: cholangiocarcinoma, ALL: acute lymphoblastic leukemia, AML: acute myeloid leukemia, EWS: Ewing sarcoma, BRC: breast carcinoma, BRDC: breast ductal carcinoma, Gli: Glioma, CRC: colorectal adenocarcinoma, ESCC: esophageal squamous cell carcinoma, GA: gastric adenocarcinoma, RCC: renal cell carcinoma, HCC: hepatocellular carcinoma, LMT: lung mesothelioma, NSCLC: non-small-cell lung cancer, SCLC: small-cell lung cancer, NHL: non-Hodgkin lymphoma, OA: ovarian adenocarcinoma, EPC: exocrine pancreatic cancer, NB: neuroblastoma, MM: multiple myeloma, Mel: melanoma, RMS: rhabdomyosarcoma, UATN: upper aerodigestive tract neoplasm, BC: bladder carcinoma, EAC: endometrial adenocarcinoma. (H) *MYC* expression as determined by RNA-seq. Adjusted P-value by DESeq2. (I) *MYC* expression as determined by quantitative RT-PCR in the indicated cell lines after *IRF2* and *BHLHE40* inactivation by CRISPR-Cas9. M1A clone 11 is a Cas9-expressing clonal derivative of 786-M1A cells. p-values calculated using one-way ANOVA. See also Figure S6.

PAX8 inhibition resistance was associated with changes in the cellular lineage state, suggesting the possibility that the role of PAX8 in supporting ccRCC growth had been replaced by alternative transcriptional lineage factors. We therefore performed a second CRISPR-Cas9 screen targeting known and predicted TFs using the P8_1_S1_1/2_(LT) cell lines (Figure 5A). As expected, constructs targeting essential genes were depleted and those targeting non-essential genes were neither enriched nor depleted (Figure S6A). We identified three new dependencies which had no phenotype in the control cells, *IRF2*, *BHLHE40*, and *ZNFX1* (Figures 5B, S6B, and S6C), all of which were expressed in cells prior to *SMARCB1* loss (Figure S6D). IRF2 is a member of a TF family which regulates Toll-like receptor signaling, hematopoietic differentiation, and the expression of interferons (IFNs) and their target genes.^32^^,^^33^ Similar to the role of PAX8 in renal development and ccRCC, IRF2 plays an important role in cancers originating from the plasma cell lineage (Figures 5C and S6E). In line with IRF2’s role in regulating IFNs, compared to P8_1_Ctrl cells, there is a strong increase in both interferon-alpha and gamma gene sets from the hallmarks collection in P8_1_S1_1/2_ cells (Figures 5D and S6F). BHLHE40 is a ubiquitously expressed stress-responsive transcription factor that is important in several physiological responses including differentiation, tumorigenesis, and response to hypoxia.^34^ The mutation of *VHL* and the stabilization of HIF2A protein is a key tumorigenic event in ccRCC, and *HIF2A* perturbation RNA-seq has placed BHLHE40 downstream of HIF2A signaling.^35^ In line with this, *BHLHE40* dependency shows tissue specificity for RCC, and P8_1_S1_1/2_ cells maintain strong HIF2A signaling when compared to P8_1_Ctrl cells (Figures 5E, S6G, and S6H). In the DepMap cohort, approximately half of the *VHL* mutant ccRCC lines are sensitive to *BHLHE40* KO, and interestingly, this includes all the *SMARCB1* mutant lines (Figure 5F). Furthermore, the dependency of ccRCC cells on *BHLHE40* anti-correlates with *PAX8* dependency (Figure S6I). ZNFX1 is a ubiquitously expressed, IFN-stimulated SF1 helicase capable of detecting viral dsRNA. Unlike *IRF2* or *BHLHE40*, dependency on *ZNFX1* is not associated with a particular lineage. Instead, there are a small number of cell lines across multiple lineages which show a strong dependency on *ZNFX1*, including TUHR10TKB, one of the three *SMARCB1* mutant ccRCC lines (Figure 5G).

PAX8 maintains ccRCC proliferative capacity by supporting the expression of *MYC*.^14^ Furthermore, compared to P8_1_Ctrl(A) cells, P8_1_S1_1/2_(LT) cells showed increased *MYC* expression levels based on our RNA-seq data (Figure 5H). This suggested that *IRF2*, *BHLHE40*, and *ZNFX1* could contribute to P8_1_S1_1/2_(LT) proliferative fitness by maintaining optimal levels of *MYC* expression. We tested this possibility by targeting *IRF2* and *BHLHE40*, the two strongest P8_1_S1_1/2_(LT)-specific hits from our TF screen (Figure 5B) using two sgRNA constructs in P8_1_S1_2_(LT) cells and a wild-type Cas9-expressing 786-M1A-derived clone (Figures S6J and S6K). As expected, IRF2 and BHLHE40 inhibition reduced the proliferation of P8_1_S1_2_(LT), but not the wild-type control cells (Figure S6L). However, *MYC* expression was activated by inhibition of IRF2 and BHLHE40 in P8_1_S1_2_(LT) cells, but not in control cells (Figure 5I). These results are in line with the observations that IRF2 and BHLHE40 can function as transcriptional repressors^36^^,^^37^ and that cancer cells require an optimal level of MYC activity for maximal proliferative capacity.^38^

### *De novo* resistance to lineage factor inhibition across cancer types

**Figure 6:**
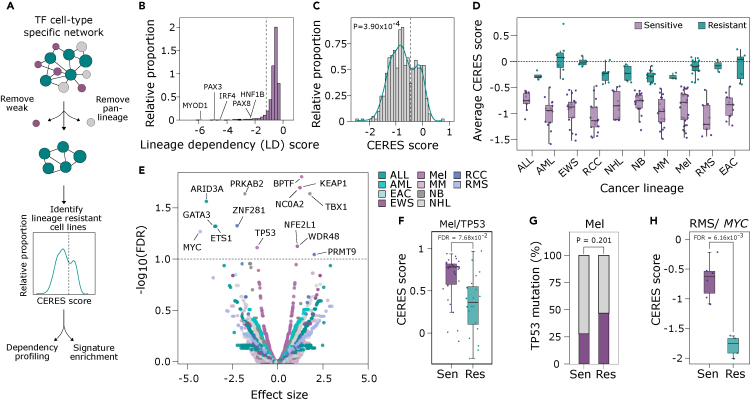
Systematic molecular definition of lineage factor inhibition resistance across cancer lineages (A) Schematic outlining the analytical pipeline used to identify lineage factor inhibition-resistant cell lines across cancer types, and uncover shared molecular features. (B) Frequency distribution of the maximum possible LD (i.e., most negative) score for each TF. For example, PAX8 has the strongest lineage-specific dependency score in RCC and so the PAX8 LD score in the RCC context is plotted here. (C) Frequency distribution of the CERES score for filtered LDs, in each cell line of their respective lineage. Distribution statistically deviated from normal determined using Shapiro Test. (D) Tukey plot of the average CERES score of each LD in their respective lineage. Sensitive and resistant lines are identified using the cutoff identified in (B) and (C), average CERES > −0.45. For boxplots, center line shows the median, the box bounds represent the first and third quartiles, and the whiskers extend to the highest and lowest values no higher of lower than 1.5 ∗ IQR. ALL: acute lymphoblastic leukemia, AML: acute myeloid leukemia, EWS: Ewing sarcoma, RCC: renal cell carcinoma, NHL: non-Hodgkin lymphoma, NB: neuroblastoma, MM: multiple myeloma, Mel: melanoma, RMS: rhabdomyosarcoma, EAC: endometrial adenocarcinoma. (E) Summary of acquired dependency analysis across different cancer lineages. FDR calculated using permutation-based statistics. Highlighted points satisfy FDR<0.1. (F) Boxplot of the CERES score of lineage-sensitive (i.e., lineage factor dependent, n = 30) and resistant (i.e., lineage factor independent, n = 19) cell lines from the melanoma lineage for *TP53* KO. FDR calculated using permutation-based statistics. For boxplots, center line shows the median, the box bounds represent the first and third quartiles, and the whiskers extend to the highest and lowest values. (G) Stacked bar plot representing the proportion of *TP53* mutations in lineage-sensitive (n = 30) and resistant (n = 19) cell lines from the melanoma lineage. p-value generated using Fisher’s exact test. (H) Boxplot of the CERES score of lineage-sensitive (i.e., lineage factor dependent, n = 6) and resistant cell (i.e., lineage factor independent, n = 4) lines from the rhabdomyosarcoma lineage for *MYC* KO. FDR calculated using permutation-based statistics. For boxplots, center line shows the median, the box bounds represent the first and third quartiles, and the whiskers extend to the highest and lowest values. See also Figure S7.

The finding that some ccRCC cell lines were insensitive to PAX8 inhibition without known inhibitory challenge on PAX8 (Figure 1I) suggested that lineage factor independence could emerge naturally during tumor evolution and that this could be associated with specific molecular features. To test this possibility systematically, we developed an analytical approach to evaluate the prevalence of lineage factor independence and its molecular determinants across different cancer lineages in large-scale CRISPR-Cas9 loss-of-function data from the cancer DepMap dataset (Figure 6A). Briefly, we identified lineage-specific TF dependencies by comparing the dependency score (CERES score) for each TF in a particular lineage against the CERES score for the same TF in cell lines pooled from all other lineages, creating a lineage dependency (LD) score for each TF in each lineage context. The distribution of the LD scores showed that for most TFs there was no specific dependency in a particular lineage, but there was a rare set of TFs which showed very strong specificity (Figure 6B). We identified specific TF dependencies in 17 of 25 lineages (LD score < (−1.2), p < 0.05) (Figure S7A).

The relative cellular dependency on individual TFs varied within lineages. In some instances, cells depended strongly on the lineage factor, for example *PAX8* in RCC, *MITF* in melanoma, and *IRF4* in multiple myeloma (MM) (Figure S7A). In contrast, the dependency on *TCF3* in acute lymphoblastic leukemia and non-Hodgkin lymphoma was considerably weaker (Figure S7A). In addition, some of the TFs with low LD scores were pan-lineage dependencies that were particularly depleted in certain lineages. These genes had a low median CERES score across all cell lines, the clearest example being *RELA* (Figure S7B); *RELA* was depleted in all 17 lineages but preferentially in MM (Figure S7C). To focus the analyses on the strongest lineage dependencies while accounting for the possibility that there are *de novo* resistant cell lines, we included genes with an overall low CERES score (≥50% of cell lines within a lineage with CERES score of ≤ −0.5, Figure S7D) and excluded pan-cancer dependencies (median CERES ≤ −0.2 across all cell lines, Figure S7E), leaving ten different lineages including hematological, epithelial, and neuroectodermal malignancies, sarcomas, and melanomas (Figure S7F and Table S7).

We then sought to uncover examples of *de novo* resistance to lineage factor inhibition. The LD scores revealed a strong bimodal distribution (Figure 6C), which was maintained at the cell line level with LD scores averaged for each cell line (Figure S7G), supporting the idea that a subset of cell lines was resistant to lineage factor inhibition. Using a distribution-informed cutoff of average CERES score > −0.45, we identified examples of lineage-resistant cell lines in all ten lineages (Figure 6D), allowing the comparison between resistant and sensitive cell lines using permutation-based statistics (Figure 6E). Interestingly, we detected instances of both acquired and lost dependencies in the lineage factor inhibition-resistant cell lines (Figure 6E). For example, in melanoma, *TP53* KO cells are enriched among *MITF*-dependent cells, in line with its known tumor suppressive role, but in *MITF*-independent cells the enrichment is less strong (Figure 6F). The reduced CERES score for TP53 in *MITF*-independent melanoma cells suggests it may already be downregulated or inactivated, thereby facilitating a transition to an *MITF*-independent state. In line with this, there is an increase in the proportion of *TP53* mutations in *MITF*-independent melanoma cell lines (47% vs. 28%) and a reduction in *TP53* mRNA expression (Figures 6G and S7H).

Other examples include rhabdomyosarcoma cells resistant to *PAX3* or both *PAX3* and *MYOD1* KO that had an enhanced dependency on *MYC* compared to lineage sensitive lines (Figure 6H). The enhanced dependency on *MYC* raises the possibility that *PAX3/MYOD1*-independent cell lines have enhanced MYC activity which promotes lineage factor resistance. In line with this, *MYC* trends toward higher expression in lineage-resistant cell lines (Figure S7I). In addition to changes in the genetic dependency profiles between resistant and sensitive cell lines, using mRNA expression data, it was possible to measure changes in the transcriptional programs. For example, differential expression analysis between lineage-resistant and sensitive neuroblastoma cell lines, coupled with gene set enrichment analysis using the hallmarks collection, revealed a very strong upregulation in the epithelial-mesenchymal transition signature (Figure S7J), paralleling the observation that a mesenchymal-like primary neuroblastoma subgroup with features of highly aggressive mesenchymal glioblastoma exists in humans.^39^ In summary, the prevalent lineage factor inhibition resistance across different cancer lineages is associated with specific molecular features and acquired genetic dependencies.

## Discussion

Transcriptional lineage factor dependencies are observed across a range of malignancies, making them an attractive target class for therapy development, but what maintains lineage fidelity in advanced cancers, and how cancer cells react to long-term lineage factor inhibition have remained unclear. We demonstrate that ccRCC cells can overcome their dependency on the renal lineage factor PAX8 through a dedifferentiation process that can be enhanced by *SMARCB1* loss. SMARCB1 maintains the kidney-specific enhancer program and its inactivation results in the loss of renal transcriptional and epigenetic identity, altering the cellular context and reducing the requirement for PAX8. Two additional SWI/SNF complex members were enriched in our screen, including ARID1A, the loss of which can facilitate hormone therapy resistance in breast cancer.^20^^,^^21^ *SMARCB1* loss can also promote hormone independence in breast cancer cells,^20^ and alterations in the SWI/SNF complex have been linked to androgen independence in prostate cancer.^40^ This suggests that resistance mechanisms to lineage-targeted therapy may converge on SWI/SNF complex members and that our findings in ccRCC may be generalizable to other cancers.

The SWI/SNF ATP-dependent chromatin remodeling complexes interact with various transcription and chromatin factors to regulate chromatin architecture and gene activation.^19^ Three distinct SWI/SNF complex subtypes with characteristic subunit complements have been described: the BRG1/BRM-associated factor complexes (BAFs), the polybromo-associated BAF complexes (PBAFs), and the non-canonical BAF complexes (ncBAFs).^41^ SMARCB1 is a member of the BAF and PBAF complexes but not of the ncBAF complex and it regulates enhancer activation states in various cell types.^31^^,^^41^ Interestingly, *SMARCB1* loss does not destabilize the BAF and PBAF complexes, but it changes their chromatin distribution through altering their interaction with nucleosomes.^42^ Enhanced lineage switching upon *SMARCB1* loss could reflect re-distribution of SWI/SNF complexes across the chromatin. More than 20% of human cancers harbor mutations in SWI/SNF complex members, but the mutation frequencies vary widely between different tumor types,^43^ highlighting the relevance of SWI/SNF complex subtype-specific mechanisms in different cancers. We find that *ARID1A* and *SMARCB1* mutations are associated with reduced *PAX8* dependency in ccRCC cell lines. As our work focused on *SMARCB1*, additional experimental analysis would be needed to test whether *ARID1A* or other SWI/SNF complex members have similar functions in lineage fidelity maintenance in ccRCC. Mutations in *ARID1A* and/or *SMARCB1* are also present in ∼5% in human ccRCCs,^44^^,^^45^ indicating that reduced lineage factor dependency as described by our results may develop naturally in some ccRCCs. Moreover, *PBRM1*, another SWI/SNF complex member, is inactivated clonally in ∼40% of ccRCCs.^6^ Possible effects of *PBRM1* loss on lineage factor activity warrants further investigation in models that recapitulate the earliest stages of ccRCC development.

Biallelic *SMARCB1* mutations are frequently observed in malignant rhabdoid tumors (MRTs) and atypical teratoid rhabdoid tumors, which are aggressive and poorly differentiated pediatric tumors that occur predominately in the kidney or soft tissue and central nervous system, respectively.^46^ However, MRTs in the kidney originate from a different cell type than ccRCCs suggesting that the molecular similarities of PAX8 inhibition-resistant ccRCC cells and rhabdoid tumors is likely to reflect the shared *SMARCB1* mutation status and general dedifferentiation rather than the acquisition of a rhabdoid ccRCC phenotype.^47^^,^^48^ In line with this, the xenograft tumors formed by PAX8 inhibition-resistant ccRCC cells did not display histological features of rhabdoid dedifferentiation.

We find that PAX8 inhibition resistance in ccRCC cells is associated with a dramatic change in tumor histology with acquired features of morphological neuroendocrine differentiation, a phenotype not commonly seen in adult renal tumors.^49^ However, even though some reports have described the expression of neuroendocrine markers in renal cancer,^50^ the tumors formed by PAX8 inhibition-resistant cells did not specifically express molecular neuroendocrine markers. Interestingly, a similar phenotype has recently been described in a mouse-derived experimental *Vhl*-mutant renal cancer model.^22^ Neuroendocrine differentiation is associated with androgen deprivation resistance in prostate cancer^51^ and EGFR inhibition resistance in lung cancer.^52^ Molecular features of neuroendocrine differentiation have also been more generally detected in a subset of different cancer types and in association with advanced disease.^53^ Neuroendocrine differentiation and morphologically similar but molecularly distinct dedifferentiation processes as described here may therefore represent a broadly shared mechanism of resistance toward different growth inhibitory insults, ranging from inhibition of hormone and oncogene signaling to direct lineage factor inhibition.

Analogous to the identification of newly acquired dependencies in lineage factor-resistant ccRCC cells, the androgen-independent state in prostate cancer and neuroendocrine differentiation more generally can result in the acquisition of new transcription factor dependencies.^53^^,^^54^ The newly acquired dependencies are unlikely to be selected purely stochastically. Rather, the high expression of IRF2, BHLHE40, and ZNFX1 already before PAX8 inhibition indicates that the cells were primed to become dependent on these factors upon lineage factor inhibition. Akin to the tissue-specific patterns of mutations in cancer, tissue-specific mechanisms are also likely to determine which acquired dependencies are most likely to emerge from lineage factor inhibition in different tissues. Our results suggest that IRF2 and BHLHE40 may contribute to the optimization of MYC levels in PAX8 inhibition-resistant cells, although other explanations for their role in maintaining proliferative fitness remain possible at this stage. An understanding of the underlying mechanisms could help predict and prevent resistance to lineage factor-targeted therapies. An alternative approach would be to target directly the possibly shared pro-tumorigenic pathways downstream of lineage factors and acquired dependencies.

Our systematic pan-cancer analysis detected frequent occurrence of *de novo* lineage factor resistance in the absence of direct lineage factor inhibition in most cancer lineages. This is supported by reports of lineage plasticity in response to multiple treatment modalities including chemotherapy, MAPK-targeted therapy, and immunotherapy, as well as environmental cues such as hypoxia and inflammation.^4^^,^^55^^,^^56^^,^^57^^,^^58^^,^^59^^,^^60^^,^^61^ We also identify shared molecular features among lineage-evaded cancer clones in several cancer types, suggesting that the development of lineage factor inhibition resistance can follow pre-determined molecular logic. However, in line with the detailed analysis of PAX8 inhibition resistance in ccRCC, resistance to lineage factor inhibition seems to develop via multiple molecular routes even within a specific lineage.

In conclusion, our study demonstrates that SMARCB1 is a key regulator of the renal enhancer program, which defines the context in which PAX8 is required for tumor growth. Resistance to PAX8 suppression can be achieved through multiple routes and it is linked to dedifferentiation, a dramatically altered tumor morphology and acquired dependencies on previously dispensable transcriptional regulators. The association between neuroendocrine differentiation and resistance to different genetic and pharmacological anticancer approaches indicates that therapeutic enhancement of lineage fidelity could be helpful in combatting acquired drug resistance in several different cancer contexts.

### Limitations of the study

Our results are based on the analysis of human cancer cell lines and xenografts. Even though the cell lines carry genetic alterations that are also commonly seen in human tumors, we cannot exclude the possibility that our observations are specific to the cell lines studied. Detailed analyses on a larger set of cell lines and human tumors would be necessary to understand how broadly applicable our results are. Our analysis of the large cancer DepMap dataset that contains hundreds of cell lines, the largest currently available dataset, revealed putative mechanisms of lineage factor independence in cell lines derived from multiple tumor types. However, larger CRISPR-Cas9 datasets would be needed for more robust interrogation of lineage factor independence across cancers. Finally, the strongly dedifferentiated histological phenotype observed in the tumors formed by PAX8 inhibition-resistant cells was not associated with the expression of neuroendocrine markers, even though it exhibited morphological features of neuroendocrine differentiation. It therefore remains unclear whether the phenotype is truly related to neuroendocrine differentiation as described in other tumor types. Current therapies do not target renal lineage factors in the clinic. The molecular consequences of lineage factor inhibition in human patients thus remain unclear at this point.

## STAR★Methods

### Key resources table

**Table:** 

REAGENT or RESOURCE	SOURCE	IDENTIFIER
**Antibodies**		

Secondary anti-mouse	DAKO	Cat# P 0447; RRID:AB_2617137
Secondary anti-rabbit	DAKO	Cat# P 0448; RRID:AB_2617138
PAX8	Santa Cruz	Cat# Sc-81353; RRID:AB_1127048
β-actin	Abcam	Cat# ab8227; RRID:AB_2305186
SMARCB1/SNF5	Bethyl laboratories	Cat# A301-087A; RRID:AB_2191714
MUC1	Abcam	Cat# ab109185; RRID:AB_10862483
SYP	Abcam	Cat# ab16659; RRID:AB_443419
NSE	Antibodies	Cat# A95480; RRID:AB_881756

**Chemicals, peptides, and recombinant proteins**		

Fugene 6	Promega	E269A
Polybrene	MiliporeSigma	TR1003G
Puromycin	Invivogen	ant-pr-1
Hygromycin	Invivogen	ant-hg-1
Blasticidin	Invivogen	ant-bl-05
RIPA lysis buffer	Sigma-Aldrich	R0278
Protease inhibitor cocktail	Sigma-Aldrich	4693132001
NuPAGE LDS sample buffer	Invitrogen	NP0007
β-mercaptoethanol	Sigma-Aldrich	444203
PVDF membrane	Millipore	IPVH00010
Immobilon Classico Western HRP substrate	Millipore	WBLUC0100
TaqMan reagents	Thermo-Fisher	4352405
Taqman probe PAX8	Thermo-Fisher	Hs00247586_m1
Taqman probe TBP	Thermo-Fisher	Hs00427620_m1
DNA ScreenTape Analysis: D1000 reagents	Agilent	5067-5583
NEBNext PCR master mix	NEB	M0541S
AMPureXP	Agencourt	A63880
KAPA HiFi HotStart ReadyMix	Roche	KK5603
Agencourt AMPure XP	Beckman-Coulter	A63880
D5000 reagents	Agilent	5067-5592

**Critical commercial assays**		

MycoAlertTM Mycoplasma Detection Kit	Lonza	LT07-318
PierceTM BCA Protein Assay Kit	Thermo-Fisher	23225
RNeasy Mini Kit	Qiagen	74004
High-Capacity cDNA Reverse Transcription Kit	Thremo-Fisher	4368814
Agilent RNA Nano 6000 Kit	Agilent	5067-1511
QuantSeq 3 mRNA-Seq Library Prep Kit FWD for Illumina	Lexogen	129-131
PCR Add-on Kit for Illumina	Lexogen	020.96
Nextera DNA library preparation kit	Illumina	FC-121-1030
minElute PCR purification kit	Qiagen	28004
QIAamp DNA mini kit	Qiagen	51304
Qubit dsDNA HS assay kit	Thermo-Fisher	Q33230

**Deposited data**		

Raw data	This study	GEO:GSE183354
Cell line dependency data, gene expression and mutation data	DepMap	https://depmap.org/portal/download/
Annotations for ENCODE candidate cis-regulatory elements for normal and cancer biosamples	SCREEN	https://screen.encodeproject.org/

**Experimental models: Cell lines**		

786-M1A	From J. Massagué, MSKCC, New York, USA	RRID: CVCL_VR30,
UOK101	From M. Linehan UOB Tumor Cell Line Repository, National Cancer Institute, Bethesda, MD	RRID: CVCL_B076

**Experimental models: Organisms/strains**		

Athymic nude mice	Charles River Laboratory	490 (Homozygous)

**Oligonucleotides**		

Non-targetting control (NTC18): GAGTGTCGTCGTTGCTCCTA	This paper	N/A
SMARCB1 (sgRNA_1): GTTCTACATGATCGGCTCCG	This paper	N/A
SMARCB1 (sgRNA_2): GTTCTACATGATCGGCTCCG	This paper	N/A
IRF2 (sgRNA_1): GCATGCGGCTAGACATGGGT	This paper	N/A
IRF2 (sgRNA_2) ACAACTTGGCAAATGTCTGG	This paper	N/A
BHLHE40 (sgRNA_1): GGGTAGGAGATCCTTCAGCT	This paper	N/A
BHLHE40 (sgRNA_2): AGACCTACAAATTGCCGCAC	This paper	N/A
Renilla (shRenilla): CAGGAATTATAATGCTTATCTA	This paper	N/A
PAX8 (sh1503): ATCCATTATTAACACAACTCTA	This paper	N/A
PAX8 (sh786): ACCGACTAAGCATTGACTCACA	This paper	N/A

**Recombinant DNA**		

Plasmid: pCW-Cas9	Eric Lander, David Sabatini	Addene ID 50661
Plasmid: LT3-Cas9-Blast	This paper	N/A
Plasmid: pKLV2-U6-gRNA(BbsI)-PGKpuro-2A-BFP	Kosuke Yusa	Addgene ID 67974
Plasmid: pKLV2-U6-gRNA(BbsI)-PGKhygro-2A-mCherry	Kosuke Yusa	Addgene ID 67977
Plasmid: LT3-eGFP-miRE-PGK-puro	Johannes Zuber	N/A
Plasmid: LT3-dsRed-miRE-PGK-Venus	Johannes Zuber	N/A
Plasmid: psPAX2	Didier Trono	Addgene ID 12260
Plasmid: pMD2.G	Didier Trono	Addgene ID 12259

### Resource availability

#### Lead contact

Further information and requests for resources should be directed to and will be fulfilled by the lead contact, Sakari Vanharanta (sakari.vanharanta@helsinki.fi).

#### Materials availability

The plasmids used in this study are listed in the key resources table. Derivatives of gifted plasmids were generated by restriction enzyme cloning. The Broad institute tool (https://portals.broadinstitute.org/gpp/public/analysis-tools/sgrna-design) was used to design sgRNAs and standard methods were used for cloning into pKLV2. Sequences for shRNA were taken from Fellmann et al., 2013^62^ and the restriction enzymes, EcoRI-HF and XhoI, were used for cloning into the miRE vectors.^62^ The materials are available from the lead contact upon request.

### Experimental model and study participant details

#### Animal studies

All animal protocols were approved by the Home Office (UK) and the University of Cambridge Animal Welfare and Ethical Review Body (PFCB122AA). Five to seven-week-old athymic female nude mice (Charles River Laboratories) were injected subcutaneously with 500 000 cells in each flank, using 100μL of 1:1 PBS/Matrigel Matrix (BD). Tumor growth was measured by calliper and tumor volume was calculated as follows, V= (length x width2) x 0.5.

#### Cell lines

The human ccRCC cell lines used were 786-M1A and UOK101. 786-M1A cells, metastatic derivatives of 786-O cells, respectively, were obtained from J. Massagué (MSKCC, New York, USA) and they have been previously described.^17^ The UOK101 cell line was obtained from M. Linehan (the UOB Tumor Cell Line Repository, National Cancer Institute, Bethesda, MD). Renal cancer cells were cultured in RPMI-1640 medium (Sigma) supplemented with 1% (v/v) penicillin-streptomycin (P/s) and 10% (v/v) fetal bovine serum (FBS). Human embryonic kidney HEK293T cells were cultured in DMEM medium (ThermoFisher Scientific) supplemented with 1% P/S and 10% FBS. Cell line identity was authenticated by short-tandem repeat profiling. Mycoplasma negativity was confirmed by the MycoAlertTM Mycoplasma Detection Kit (Lonza, LT07-318) or by qRT-PCR (PhoenixDx® Mycoplasma Mix).

### Method details

#### Drug treatment

Doxycycline (Sigma) was diluted in RPMI media to a final concentration of 0.1, 0.3, 0.6, or 1μg/ml (as specified in results) from a stock concentration of 1mg/ml, before adding to the cells. Doxycycline infused media was replenished every 2-3 days depending on the length of the treatment.

#### Lentiviral transduction

The plasmid of interest and the viral packaging plasmids psPAX2 and pMD2.G were co-transfected into HEK293T cells using Fugene 6 (Promega E269A) according to the manufacturer’s protocol. Viral supernatants were harvested 48h following transfection and filtered through a 0.45μM PVDG sterile filter. One day prior to transduction 2.5×10^5^ cells were seeded on a 6-well plate. Immediately before transduction, the medium was changed to RPMI containing 6-8μg/mL Polybrene (Millipore). Fresh or frozen viral supernatant was added to the cells accordingly. 24h after transduction the media was changed to fresh RPMI. Antibiotic selection media were added two days post-transduction: 4μg/ml puromycin (Invivogen), 800μg/ml hygromycin (Invivogen) or 25μg/ml blasticidin (Invivogen).

#### Immunoblotting

Cell pellets were washed once with ice-cold PBS and lysed with RIPA lysis buffer (Sigma) containing 1x protease inhibitor cocktail (Sigma): cells were incubated on ice for 30m and vortexed every 10m, followed by centrifugation at 14,000 RPM at 4°C. The protein lysate (supernatant) was collected and quantified using the PierceTM BCA protein assay kit (Thermo Scientific), before being stored at -80°C. For immunoblotting, 10μl of protein (15-30μg) was added to 4μl of loading buffer (Invitrogen, NuPAGE) and 1 of β-mercaptoethanol (Sigma) and topped up to a total volume of 20μl with H_2_O. Samples were boiled at 95°C for 5m and then centrifuged for 20s. Following centrifugation, samples were loaded onto a polyacrylamide gel alongside a precision plus protein standards kaleidoscope ladder (BIO-RAD) and run at 80V for 15m followed by 100V for 2h. Proteins were transferred onto a PVDF membrane (Millipore) for approximately 2-3h at 100V. The membrane was subsequently blocked in 5% milk (dissolved in 0.1% PBS-Tween, PBST) for 1h. For immunoblotting, primary antibodies were diluted in 5% milk and incubated overnight with the membrane at 4°C. Membranes were washed three times with PBST before incubation with the secondary anti-mouse (DAKO, cat no. P 0447, 1:10,000) or anti-rabbit antibodies (DAKO, cat no. P 0448, 1:5000) conjugated to horseradish peroxidase (HRP) for 2h. Membranes were washed three times with PBST and were developed with LuminataTM Classico Western HRP substrate (Millipore) using a film processor. The following primary antibodies were used: PAX8 (Santa Cruz, cat no. Sc-81353.,1:250), β-actin (Abcam, cat no. ab8227, 1:20,000), SMARCB1/SNF5 (Bethyl laboratories, cat no. A301-087A, 1:2500).

#### Immunohistochemistry

Tumor xenografts were collected and fixed overnight in 4% paraformaldehyde, washed, embedded in paraffin, and sectioned. MUC1 (Abcam, ab109185, 1:500), SYP (Abcam, ab16659,1:200) and NSE (Antibodies, A95480, 1:100) staining was performed in a Bond-Max instrument (Leica) using Bond Polymer Refine Detection reagents (Leica) according to the manufacturer's protocol (IHC Protocol F).

#### Reverse transcription and PCR

Cells were pelleted at 500g for 3m and stored at -80°C before processing. RNA was extracted using the RNeasy Mini Kit (Qiagen), according to the manufacturer’s instructions. Reverse transcription-PCR was performed using the High-Capacity cDNA Reverse Transcription Kit (Thermo Scientific) to generate cDNA from 500ng of RNA. The StepOnePlusTM Real-Time PCR instrument (Thermo Scientific) was used with TaqMan reagents (Thermo Scientific). Samples were run in triplicate, normalized to the housekeeping gene TATA-box binding protein (TBP), and analyzed using the double delta Ct method. Taqman probes used: PAX8 (Hs00247586_m1) and TBP (Hs00427620_m1).

#### *In vitro* proliferation assays

6 x 10^3^ 786-M1A cells were seeded in triplicate on a 24-well cell culture plate and analyzed using the IncuCyte ZOOMTM instrument (Essen Bioscience). Bright-field images were acquired in 9 independent locations within each well every 2 hours. Confluency was measured by applying a predefined cell-specific mask to each image, which distinguished the cells from the background. For competition assays, dsRED/eGFP was used to gate all cells expressing an shRNA, and BFP/eGFP/mCherry was used to measure the abundance of two co-cultured cell populations. The proportion of the competing cell populations was measured by flow cytometry on an LSR Fortessa (BD Biosciences) and compared to day 0. The following gating approach was used: FSC-A, FSC-W, SSC-A to distinguish single cells from debris, and then dsRed (561nm, 610/20nm), mCherry (532nm, 610/20nm), BFP (405nm, 450/50nm) or GFP (488nm, 515/20nm), venus (488, 530/30) channels for discriminating between cell populations.

#### Fluorescence-activated cell sorting and analysis

Fluorescence-activated cell sorting was performed using a BD LSRFortessa flow cytometer. FlowJo software (BD Biosciences) was used to analyze flow cytometry data and generate plots (Figure S8). Fluorescence-activated cell sorting was carried out by the Flow Cytometry Core Facility at the Cambridge Institute for Medical Research.

#### RNA-sequencing

A total of four replicates per condition in 6 well plates were seeded 24 hours before library preparation. The cells were lysed on ice in buffer RLT (RNeasy Plus Mini Kit Qiagen). Total RNA was extracted using the RNeasy Mini Kit (Qiagen), according to the manufacturer’s instructions. RNA quality was determined using Agilent RNA Nano 6000 kit (Agilent 5067-1511) and RNA concentration was determined using a NanoDrop 1000 Spectrophotometer. Library preparation was performed using the QuantSeq 3 mRNA-Seq Library Prep Kit FWD for Illumina and PCR Add-on Kit for Illumina (Lexogen), with 300ng input RNA. The size of the final libraries was determined using the Agilent 4200 TapeStation System using the high sensitivity D1000 reagents (5067-5592). The concentration of the libraries was determined using the Qubit Flex Fluorometer (Thermo Fisher). The libraries were pooled in equimolar concentrations and submitted for deep sequencing on the Illumina HiSeq4000 platform (SE50).

#### RNA-sequencing analysis

The processing of the FASTQ files to a read count table was performed on the BlueBee® Genomics Platform. Reads were trimmed using Bbduk (v35.92) from the bbmap suite, aligned with STAR aligner (v2.5.2a) to hg38, and counted using HTSeq-count (v0.6.0). Differentially expressed (DE) genes were determined from the read counts table using DESeq2 (v1.26.0).^63^ The custom rhabdoid signature was generated using common DE genes upon SMARCB1 re-introduction in TTC1240 and G401 cell lines, which satisfied log2FC < -0.5 and padj < 0.05.^31^ The top 500 up and down-regulated genes with a padj < 0.05 upon SMARCB1 KO were used to generate gene signatures for validation with CCLE expression data. Gene set enrichment analysis with custom and mSigDB (v7.2.1) signatures was performed using the R package ClusterProfiler (v3.14.3).^64^ Data wrangling and presentation (MA plots) were achieved using the R packages Tidyverse (v1.3.0) and ggpubr (v0.4.0), respectively.

#### ATAC-sequencing

786-M1A cells were treated with 0.6μg/ml doxycycline for 6 days before harvesting at 70% confluency. On the day of harvest, cells were trypsinized, counted, nuclei extracted, and 50,000 cells were used for the ATAC-seq protocol.^65^ ATAC libraries were generated with the Illumina Nextera DNA library preparation kit (FC-121-1030) and purified for amplification with the minElute PCR purification kit (Qiagen 28004). The libraries were amplified for a total of 8-12 cycles using custom Nextera PCR primers and NEBNext PCR master mix (NEB M0541S). The amplified libraries were purified using Agencourt AMPureXP reagents (A63880), profiled on the Agilent 4200 TapeStation System using the high sensitivity D5000 reagents (5067- 5589), pooled in equimolar concentrations, and submitted for sequencing on the Illumina HiSeq4000 platform (SE50).

#### ATAC-sequencing analysis

Adapters and low-quality bases (quality < 20) were trimmed from read ends using cutadapt (version 2.10). Reads were mapped to hg38 using BWA (version 0.7.17). Low quality reads (mapping quality < 20) and reads mapping to ENCODE blacklisted regions and regions other than chr1-22, chrX and chrY were removed using deepTools2.^66^^,^^67^ Reads were corrected for Tn5 offset (+ve strand: +4bp, -ve strand: -5bp). Peaks were called using MACS2 (version 2.2.7.1) with the following parameters “-f BAM –bdg -g 2913022398 –nomodel –nolambda –shift -100 –extsize 200”.^68^ A consensus peak file for DE analysis was generated by extending peak summits to a fixed 501bp window (using BEDtools v 2.30.0), ranking called peaks by their qvalue, and iterating down the list, removing any overlapping peaks with a lower qvalue. This produced a consensus peak file containing the coordinates of the most significant peak called at a particular locus. The read count table was generated by extending reads to the modal length of 250bp and counting the number of uniquely mapped reads falling within consensus peaks using RSamtools (v2.2.3). Peaks were filtered for –log10(q)<20 and differentially accessible (DA) peaks (FC+/-2 and padj<0.001) were determined using DESeq2.^63^ Homer (v4.11) was used for *de novo* and known motif enrichment analysis on +/- 50bp flanking the summits of DA regions, compared to a set of high confidence unchanged regions.^69^ The R package ChIPseeker (v1.22.1) was used to determine genomic annotations for peaks. To determine the correlation between gene expression and epigenetic changes, sequentially larger windows around LA and HA peaks were created with GenomicRanges (v1.38.0), and a hypergeometric based test (pHyper) was used to determine whether genes captured within these windows were significantly enriched for down or up-regulated genes, respectively. EAseq (v1.111) was used to determine genes that fell within these windows (GColoc function) and to create a set of matched controls (Controls function).^70^ For data visualization, EnhancedVolcano (v.1.4.0) was used for volcano plots and EAseq (v1.111) was used for genomic tracks, heatmaps, and metagene plots.^70^

#### Pooled CRISPR-Cas9 screening

Cells were transduced with a lentiviral library at a low MOI (<0.3) to ensure 1000x sgRNA representation. An MOI of <0.3 was used so that >85% of cells had a single sgRNA integration. After 48h following transduction, the cells expressing the integrated library were selected for with puromycin or hygromycin for 5 days. For doxycycline naive cells, the screen was initiated after antibiotic selection by supplementing the medium with 0.6μg/ml doxycycline to induce the expression of Cas9, otherwise, the screen was considered to have started 24h post-transduction. Cells were cultured for 17-21 days after screen initiation and two replicates at various time points were collected for each condition. For time points that required FACS, enough cells to ensure >130x coverage were harvested, otherwise, >500x coverage was maintained. Day 17 of the chromatin regulator screen was the only time point that required sorting. Given that the primary focus of this screen was to look for enriched hits and that the initial coverage was ∼1000x, an sgRNA coverage of 130x was tolerated. Genomic DNA was isolated using the QIAamp DNA mini kit (Qiagen 51304) and libraries were created by amplifying the cassette containing the sgRNAs using KAPA HiFi HotStart ReadyMix (Roche) and custom primers^71^ adapted for pKLV2. Libraries were purified using Agencourt AMPure XP (Beckman-Coulter A63880) beads, profiled using the Agilent 4200 TapeStation System using the high sensitivity D1000 reagents (5067-5592), quantified using the Qubit dsDNA HS assay kit (Thermo) and pooled in equimolar concentrations for sequencing on the Illumina HiSeq4000 platform (SE50). FASTQ files from sequencing were aligned to the sgRNA library and counted, using the command mageck count (v0.5.9), which tolerated no mismatches. Raw fold changes from normalized counts were calculated for each sgRNA construct for each gene, compared to Day 0 or the plasmid library. The top performing 3 sgRNAs (depleted or enriched depending on context) for each gene averaged across the experimental screen arm (i.e. P81/2 for the TF screen) were used to calculate the final beta scores (normalized FC) for each gene. Beta scores were calculated using mageck mle (v0.5.9) with the normalization method set to median and permutations set to 1000.

#### The encyclopedia of DNA elements (ENCODE) analysis

DNAse I hypersensitivity profiles for cell lines (n=97), adult primary cells and tissues (n=125), and embryonic tissues (n=282) were downloaded from SCREEN (https://screen.encodeproject.org/), with chromatin accessibility annotations (open/closed) determined for each of the 926,535 candidate cis-regulatory regions (cCREs) identified by the ENCODE project. For clustering, the top 250,000 most variable regions across samples were selected to generate a pairwise Pearson correlation matrix. To identify clusters, samples were first ranked based on the number of other samples with which they highly correlated (PCC>0.6). Starting with the top-ranked sample, all samples which correlated (PCC>0.6) were identified and labelled as cluster 1. These samples were then removed from the matrix, the matrix was re-ranked and cluster 2 was identified by the same means. This process was repeated until the size of the cluster dropped below a cut-off of five samples. This resulted in 21 clusters, incorporating 376/504 samples. Clusters were given a biological annotation based on their sample composition, and clusters which could not be annotated were removed (n=1). Cluster-specific cis-regulatory regions were defined as peaks that were present in 80% of cluster samples and appeared in no more than two additional clusters. Overlap analysis between cluster peaks sets and +/-25bp flanking the summit of DA regions from this study was performed using the R package ChipPeakAnno, requiring a stringent 100% overlap.

#### Cancer cell line encyclopedia (CCLE)

Mutational and gene expression data were downloaded from the DepMap portal (https://depmap.org/portal/download/). Differential expression analysis with raw RNA-seq counts was performed as above. Biallelic inactivation of the essential gene *VHL* is a truncal initiation event in ccRCC, RCC cell lines were considered ccRCC if they had a ‘damaging’ or ‘other non-conserving’ mutation in *VHL*, which also corresponded with resistance to *VHL* KO (CERES > -0.5). To prioritize functionally relevant mutations in *ARID1A* and *SMARCB1*, ‘damaging’ or ‘other non-conserving’ with a TCGA or COSMIC hotspot were considered.

#### Pan-cancer lineage dependency (LD) analysis

Genome-wide CRISPRcas9 genetic dependency data for 946 cell lines was downloaded from the DepMap project (www.depmap.org/portal/).^72^^,^^73^ The list of ∼18,000 genes was filtered for ∼1,600 TFs, as defined by Lambert et al.^74^ The Lineage of a cell line was defined by their lineage annotation (e.g., skin) and lineage subtype annotation (e.g., melanoma). Of the 81 defined lineages (e.g., skin_melanoma), 56 were removed because they were underrepresented (<10 cell lines). A lineage dependency score (LD_score_) for each TF in each lineage context was calculated according to: LD_score_ = mean(CERES)_lineage x_ - mean(CERES)_remaining_ _lineages_. The LD_score_ was a measure of how specific a particular transcriptional dependency was to a certain lineage context; a larger negative score denotes a stronger and more specific dependency. Simultaneously, the Kruskal-Wallis statistical test in conjunction with a Benjamini Hochberg correction was implemented to derive a corresponding p value for each LD_score_. To define putative core regulatory circuitry (CRC) for each of the 25 lineages, three levels of filtering were applied. (1) Based on the distribution of maximum LD_scores_ for each TF, a cut-off of LD_score_ <-1.2 and a *p*-value < 0.05 was implemented. The distribution of maximum LD_scores_ was plotted by selecting the lowest possible LD_score_ for each TF. For example, PAX8 and HNF1B had the most negative LD_score_ in the RCC lineage context, and so these scores were used. (2) Examples of LDs that were strong cellular dependencies were selected by filtering for putative LDs for which the majority (>50%) of cell lines in their respective lineage had a CERES score of ≤ -0.5. (3) Putative LDs which were pan-cancer dependencies but were more strongly depleted in a particular lineage were also removed. This was accomplished by using a plot of the distribution of the median CERES score across all cell lines for each putative LD. Based on the bimodal distribution of the data, a cut-off medianCERES >-0.2 was identified (Figure S7E). After filtering, CRC predictions were available for 10/25 lineages. To identify lineage-resistant cell lines within each of the 10 lineages, a distribution of the averaged CERES scores of LDs in each cell line of their respective lineage was plotted (Figure 6C). Based on the bimodal distribution of the data, a cut-off of average CERES score > -0.45 was used to identify lineage-resistant cell lines.

A permutation-based statistical method was used to identify acquired transcriptional dependencies or lack of dependencies in lineage resistant versus lineage sensitive lines within each lineage. (1) Compute the observed effect size using Cohen’s D (lineage resistant vs lineage sensitive) relative to each TF. (2) Create a permuted dataset by randomly dividing cell lines into sensitive and resistant whilst maintaining the original number of observations for each of the two categories. (3) Compute the effect size with the permuted data. (4) Repeat steps 2 and 3 1000 times. (5) For each feature *i*(*i* = 1,…,*n*), compute the FDR associated to its observed effect size:

$$F\hat{D}R_{i}=\frac{expected\;number\;of\;false\;positives}{number\;of\;true\;positives}=\frac{E\left[\sum_{j=1}^{n}I\left(\left|x_{j}^{perm}\right|\geq \left|x_{i}^{obs}\right|\right)\right]}{\sum_{j=1}^{n}I\left(\left|x_{j}^{obs}\right|\geq \left|x_{i}^{obs}\right|\right)}$$

The numerator is the mean (over the 1000 permutations) number of false positives (features with absolute effect size greater than or equal to the absolute observed effect size of feature). The denominator is the number of observed features with absolute effect size greater than or equal to the absolute observed effect size of feature. (6) The q value associated to each feature was computed as:

$$q-{value}_{i}=\min_{j\;s.t.\;\left|x_{j}^{obs}\right|\geq \left|x_{i}^{obs}\right|}F\hat{D}R_{j}$$

and a threshold of 0.1 was used to call significant features.

### Quantification and statistical analysis

Statistical analyses were performed in R. The Kruskal-Wallis test was used for competitive proliferation assays and comparison of dependency data between subtypes. For Kaplan-Meier curves of tumour free progression, the logrank test was used. The hypergeometric distribution (phyper) test was used to measure significance of ATAC/DNAse I, gene set, and genomic region overlaps. Pearson correlation was used for correlation analysis. The Wilcoxon test was used for gene expression comparison. For all tests, a p-value of <0.05 was considered significant.

## Data Availability

• All RNA-seq and ATAC-seq files have been made available via the Gene Expression Omnibus and are publicly available as of the date of publication. Accession numbers are listed in the key resources table. This paper analyzes existing, publicly available data. Public resources that have been analyzed in this paper are listed in the key resources table. • The code used in this paper and related information are available from the lead contact upon request. • Any additional information required to reanalyze the data reported in this paper is available from the lead contact upon request.

## Supplemental information

Document S1. Figures S1–S8

Table S1. DESeq2 analysis Ctrl.Ctrl_vs._P81Ctrl

Table S2. DESeq2 analysis Ctrl.Ctrl_vs._P81S112(A)

Table S3. DESeq2 analysis Ctrl.Ctrl_vs._P81S112(LT)

Table S4. DESeq2 analysis P81Ctrl_vs._P81S112(A)

Table S5. DESeq2 analysis P81Ctrl_vs._P81S112(LT)

Table S6. DESeq2 analysis P81S112(A)_vs._P81S112(LT)

Table S7. Filtered lineage-specific TFs
