# Supplementary material for: The SWI/SNF complex member SMARCB1 supports lineage fidelity in kidney cancer
Source: iScience. 2023 Jul 13;26(8):107360. doi: 10.1016/j.isci.2023.107360 (PMC10405256; doi:10.1016/j.isci.2023.107360)
Supplement: Document S1. Figures S1–S8 [file mmc1.pdf]

iScience, Volume *26*

Supplemental information

The SWI/SNF complex member SMARCB1 supports lineage fidelity in kidney cancer

Ludovic Wesolowski, Jianfeng Ge, Leticia Castillon, Debora Sesia, Anna Dyas, Shoko Hirosue, Veronica Caraffini, Anne Y. Warren, Paulo Rodrigues, Giovanni Ciriello, Saroor A. Patel, and Sakari Vanharanta

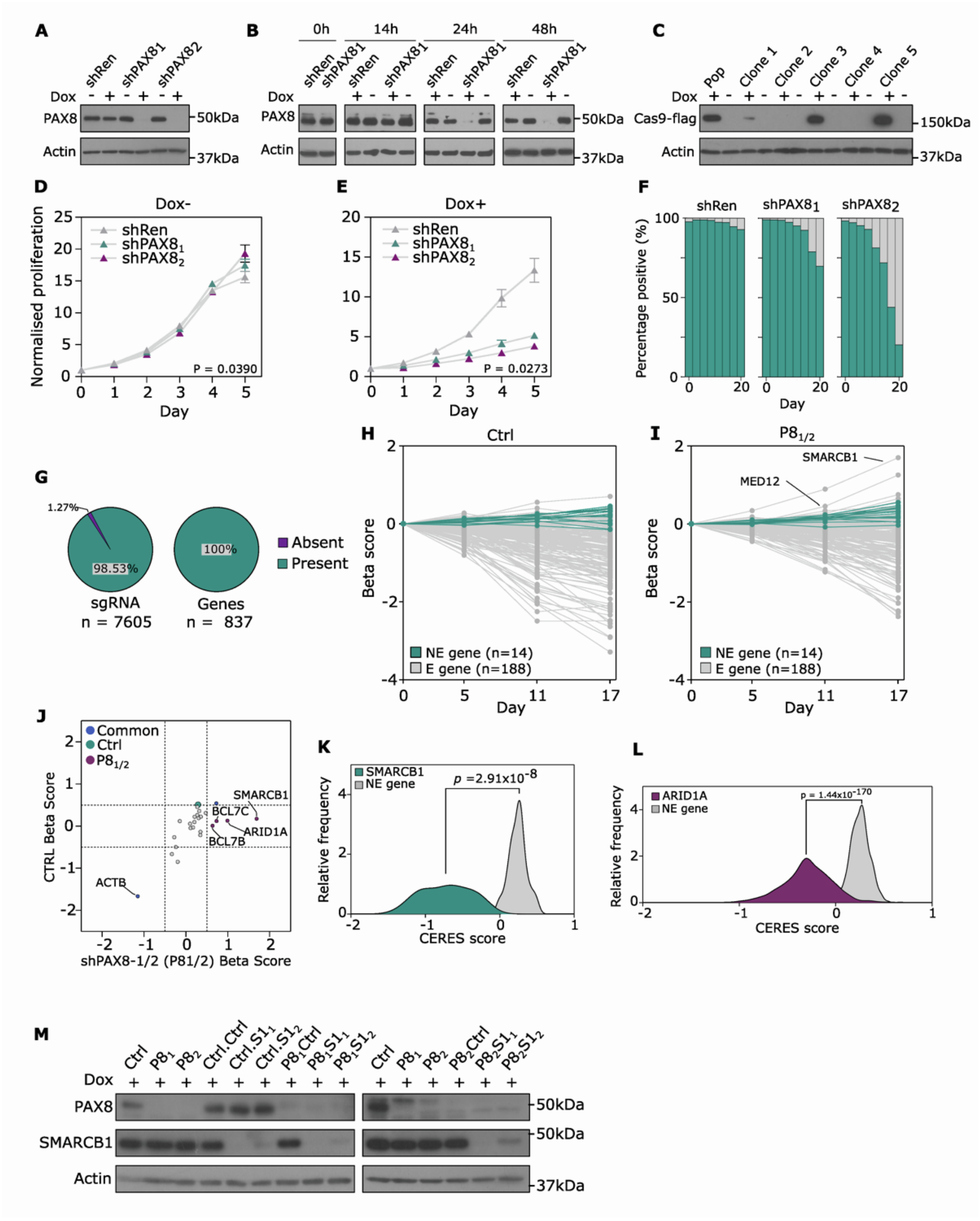

Figure S1. Functional consequences of PAX8 inhibition in ccRCC cells, related to Figure 1. (A) Western blot of PAX8 expression in 786-M1A cells expressing doxycycline-inducible shRNAs targeting PAX8 ($PAX8_{1/2}$) or a negative control (shRen). Cells were treated with 0.6µg/ml doxycycline for three days. **(B)** Western blot time course of PAX8 expression in 786-M1A cells expressing doxycycline-inducible shRNAs. Cells were treated with 0.6µg/ml doxycycline. **(C)** Western blot analysis of

Cas9 expression in 786-M1A doxycycline-inducible Cas9 single-cell clones. Cells were treated with 0.6µg/ml doxycycline for 3 days. **(D-E)** Confluency-based cellular proliferation assay, with 786-M1A Cas9 clone 6 (786-M1A-C6) cells expressing inducible shRNAs. Cells were pre-treated with 0.6µg/ml doxycycline for 3 days before assay start. Three technical replicates per condition. Error bars are SD. Kruskal-Wallis test. **(F)** Escaper assay with 786-M1A-C6, showing the percentage of cells expressing the fluorophore dsRed as a measure of shRNA expression. **(G)** Pie chart of percentage sgRNA constructs and genes represented at day 0 of the chromatin regulator CRISPR-Cas9 screen. **(H-I)** Beta scores of essential (n=188) and non-essential genes (n=14) at time points throughout the screen for the control arm (H) and experimental arm (I). **(J)** Changes in sgRNA abundance over time, measured by calculating beta scores using the top three enriched sgRNAs per gene relative to day 0, from two technical replicates. The beta scores for the control arm of the screen versus the pooled experimental arm, filtered for SWI/SNF complex members. Highlighted points have a beta score <-0.5 or >0.5 and a p-value < 0.05. *P*-value was calculated by permutation-based approach using MAGeCK. **(K)** Genetic dependency data for renal cell carcinoma lines (n=21) from the DepMap project. Distribution of CERES scores for *SMARCB1* and an example non-essential gene (*CNBD1*). Kruskal-Wallis test. **(L)** Genetic dependency data from the DepMap project (n=946). Distribution of CERES scores for *ARID1A* and an example non-essential gene (*CNBD1*). Kruskal-Wallis test. **(M)** Western blot analysis of PAX8 and SMARCB1 expression in 786-M1A-C6 cells expressing combinations of sgRNAs (ctrl or $S1_{1/2}$) and inducible shRNAs (ctrl or $P8_{1/2}$). Cells were treated for 6 days with 0.6µg/ml doxycycline. NE: non-essential. E: essential.

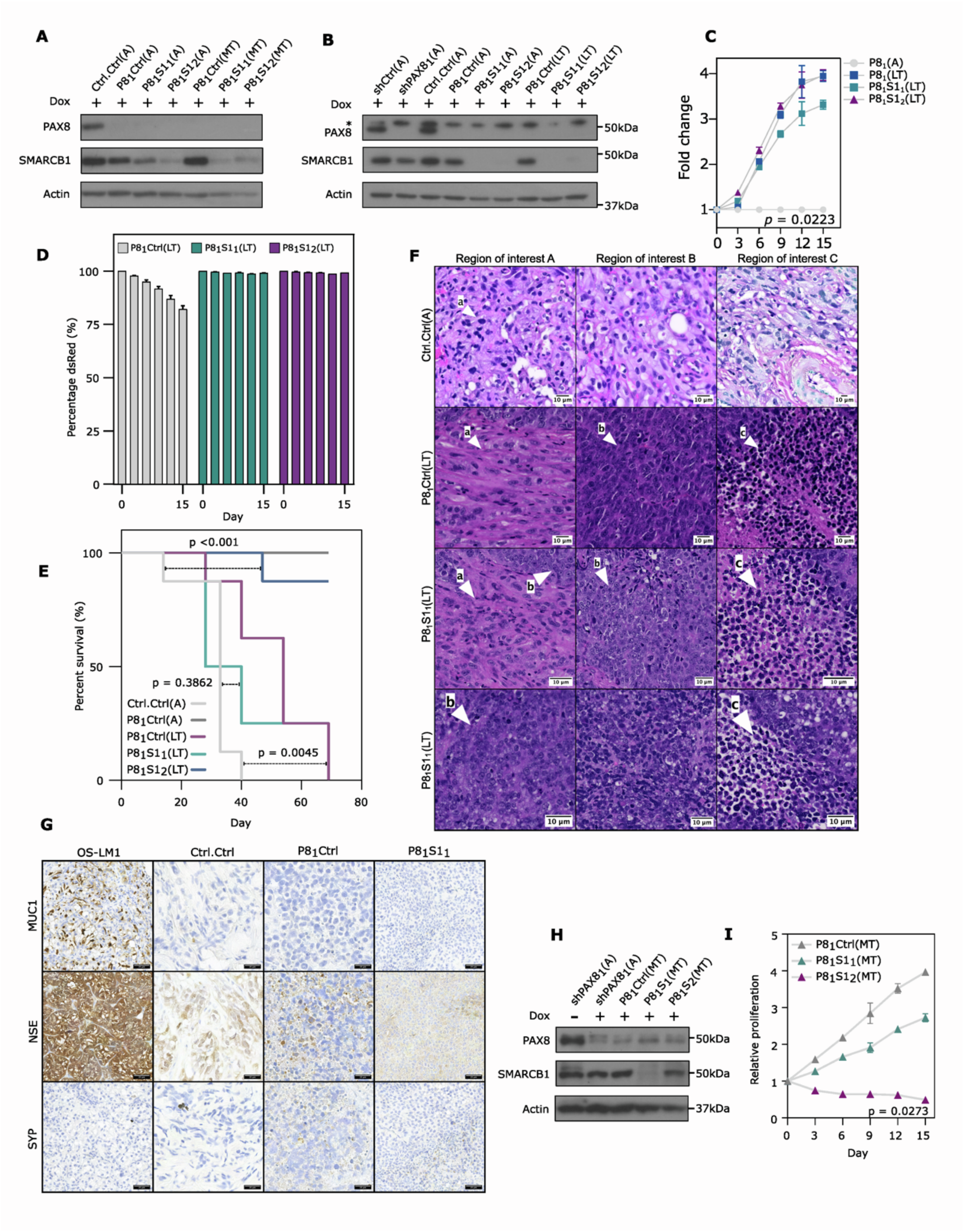

Figure S2. Characterization of lineage factor inhibition resistant ccRCC cells, related to Figure 1. (A-B) Western blots of PAX8 and SMARCB1 expression for 786-M1A-C6 cells expressing combinations of sgRNAs (Ctrl or $S1_{1/2}$) and inducible shRNAs (Ctrl or $P8_1$), cultured for (A) ~1month (MT) and (B) ~2-3 months (long term, LT) on 0.6μg/ml doxycycline, compared to acutely (A) treated cells. The asterisk on

the PAX8 plot denotes an unspecific background band. **(C)** Competitive proliferation assay with 786-M1A-C6 cells expressing combinations of sgRNAs (ctrl or $S1_{1/2}$) and inducible shRNAs (ctrl or $P8_{1/2}$), cultured for ~2-3 months (LT) on 0.6μg/ml doxycycline, competed against cells with a PAX8 KD. To establish a PAX8 KD to compete against, doxycycline was added 3 days prior to starting the competition assay. Error bars are SD. Three technical replicates per condition. Kruskal-Wallis test. **(D)** Escaper assay showing the percentage of 786-M1A-C6 cells expressing the fluorophore dsRed as a measure of PAX8 shRNA expression for cells pre-cultured on doxycycline for ~2-3 months (LT), normalized to day 0. Cells were sorted at the beginning of the assay to ensure a starting point of 100% dsRed. Two biological replicates, error bars are SD. **(E)** Kaplan-Meier analysis of tumor-free survival in athymic mice, after subcutaneous injection of the following 786-M1A-C6 cell lines; Ctrl.Ctrl(A), $P8_1$Ctrl(A), $P8_1$Ctrl(LT), $P8_1S1_1$(LT) and $P8_1S1_2$(LT). Acute cells were treated with 0.6μg/ml doxycycline for 3 days prior to injection. Long term cells were maintained on 0.6μg/ml doxycycline. Four mice were injected with $5x10^5$ cells in both flanks per condition. Logrank test. **(F)** Hematoxylin and eosin (H&E) staining of tumors from (E). Tumors were harvested at day 47 after subcutaneous injection. Examples of high-grade ccRCC with sarcomatoid dedifferentiation, high-grade undifferentiated histology with morphological neuroendocrine differentiation and regions of necrosis are marked as a,b,c respectively. **(G)** Immunohistochemistry using antibodies targeting neuroendocrine markers in the indicated tumors. Xenograft tumors formed by OS-LM1 cells, a *VHL* mutant cell line that forms tumors with a clear cell phenotype, used as a control. **(H)** Western blot of PAX8 and SMARCB1 expression in UOK101 cells, expressing a combination of sgRNAs (ctrl or $S1_{1/2}$) and an inducible shRNA targeting PAX8 ($P8_1$). Acute (A) cells were pre-treated with 0.6μg/ml of doxycycline for

three days and midterm (MT) cells were cultured on 0.6µg/ml doxycycline for ~1month. **(I)** Competitive proliferation assay with UOK101 cells expressing a combination of sgRNAs (ctrl or $S1_{1/2}$) and a PAX8 inducible shRNA ($P8_1$), cultured for ~1 month on doxycycline (MT), competed against cells with a PAX8 KD. To establish a PAX8 KD to compete against, doxycycline was added 3 days before starting the assay. Three technical replicates per condition. Error bars are SD. Kruskal-Wallis test.

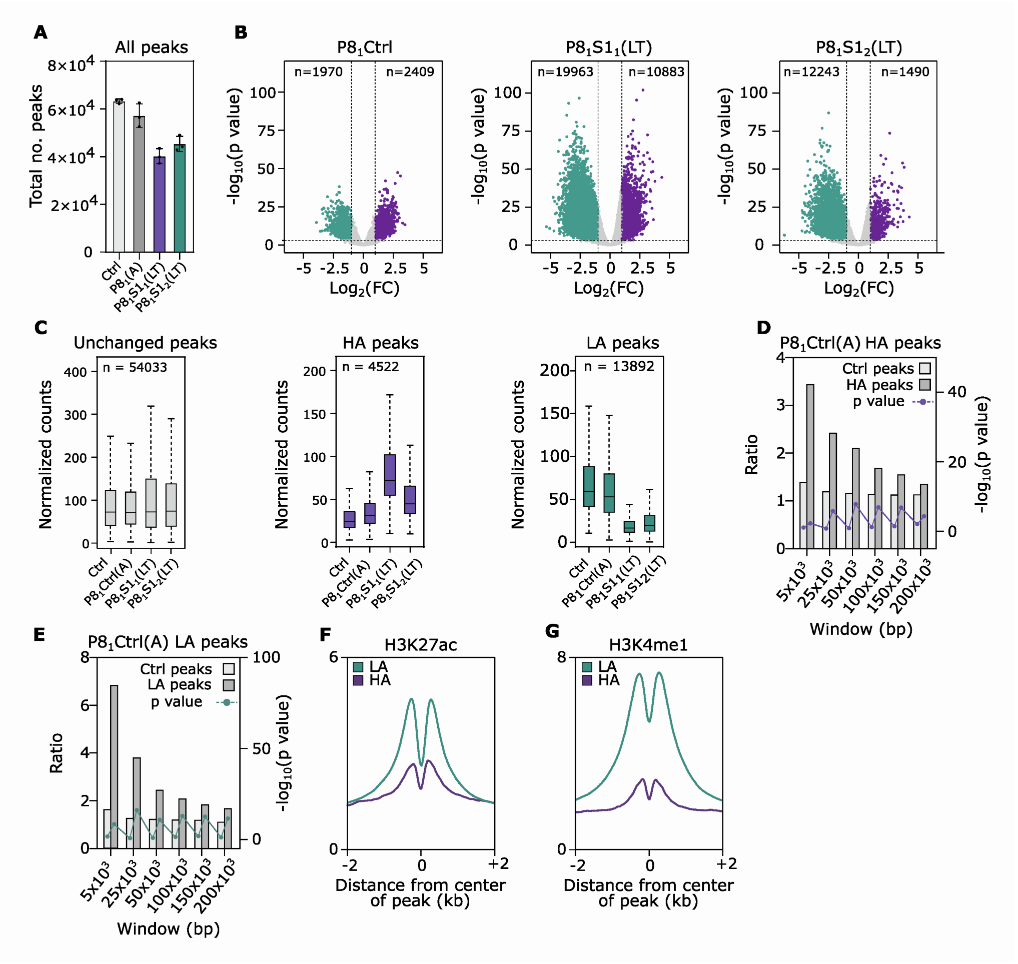

Figure S3. Chromatin alterations in lineage factor inhibition resistant ccRCC cells, related to Figure 2. (A) Total number of ATAC-seq peaks from the consensus list of all peaks merged across conditions (n= 72 447) called for each condition. Three technical replicates per condition. Error bars are SD. **(B)** Volcano plots showing differentially accessible ATAC-seq regions for Ctrl.Ctrl(A) vs P8$_1$Ctrl(A), P8$_1$S1$_1$(LT) and P8$_1$S1$_2$(LT). Highlighted points satisfy FC >2 or <(-2) and p.adjust < 0.001. Adjusted *p*-value calculated with DEseq2. **(C)** Tukey plot of normalized counts across samples from (A) for high and low accessibility peak sets and all remaining peaks from the consensus list of all peaks merged across conditions, defined by Ctrl.Ctrl vs P8$_1$S1$_{1/2}$ (LT) (FC >2 or <(-2), p.adjust <0.001). For boxplots, center line shows the median, the box bounds represent the first and third quartiles and the whiskers extend

to the highest and lowest values, no further than 1.5 × IQR. **(D-E)** Correlation of ATAC-seq peak and transcriptional changes, for Ctrl.Ctrl(A) vs $P8_1$Ctrl(A). Left y-axis, the ratio of the number of down/up-regulated genes found within windows created around lower/higher accessible regions compared to the number of expressed genes (universe) also found within the windows. Left y-axis, *p*-value, hypergeometric test. Matched Ctrl peaks for lower and higher accessible regions were generated from the consensus list of all peaks merged across conditions. **(F-G)** Average H3K27ac (F) and H3K4me1 (G) signal for higher accessible and lower accessible region sets in control 786-M1A cells reanalyzed from Rodrigues et al.[25]

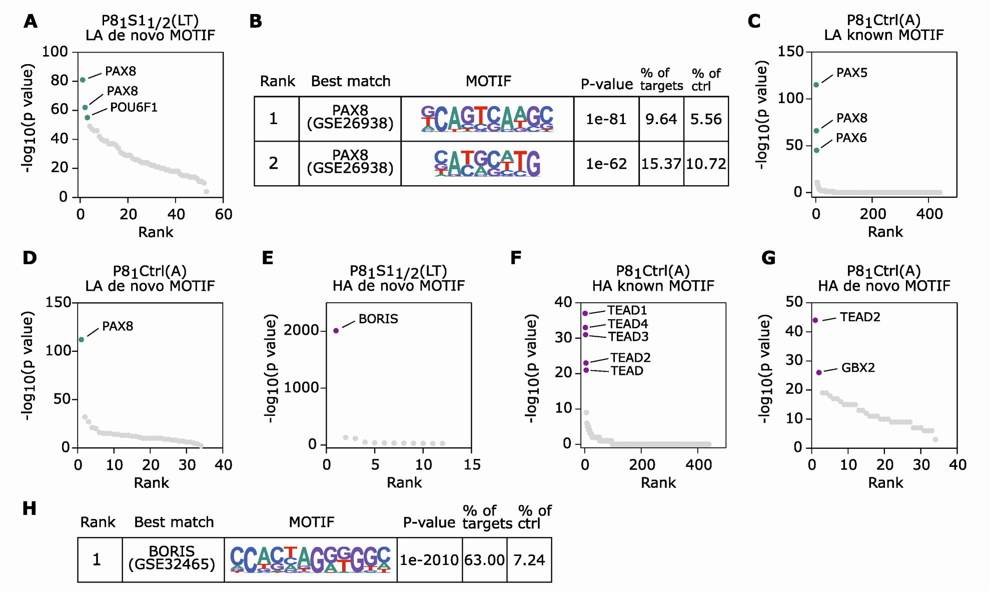

Figure S4. DNA motif analysis on the open chromatin regions of lineage factor inhibition resistant ccRCC cells, related to Figure 3. (A) Ranked plot of *de novo* DNA motif analysis for lower accessible regions from Ctrl.Ctrl(A) vs $P8_1S1_{1/2}$(LT). *P*-value calculated using Homer **(B)** Highest two scoring *de novo* motifs from (A). **(C-D)** Ranked plots of (C) known and (D) *de novo* DNA motif analysis for lower accessible regions from Ctrl.Ctrl(A) vs $P8_1$Ctrl(A). *P*-value calculated using Homer. **(E)** Ranked plot of *de novo* DNA motif analysis on higher accessible regions from Ctrl.Ctrl(A) vs $P8_1S1_{1/2}$(LT). *P*-value calculated using Homer. **(F-G)** Ranked plots of (F) known and (G) *de novo* DNA motif analysis for higher accessible regions from Ctrl.Ctrl(A) vs $P8_1$Ctrl(A). *P*-value calculated using Homer. **(H)** Highest scoring *de novo* motif from (E).

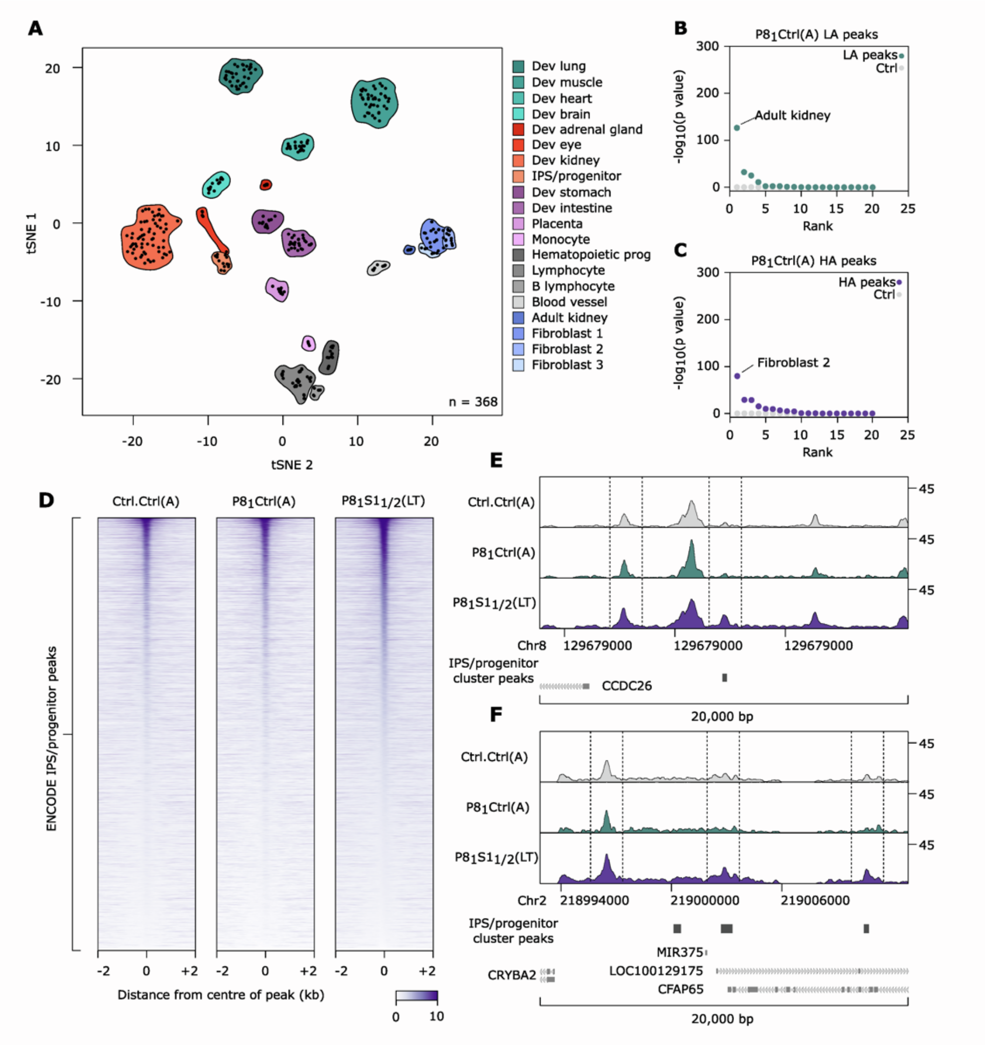

Figure S5. Changes at developmental enhancers in lineage factor inhibition resistant ccRCC cells, related to Figure 3. (A) tSNE plot based on the top 250,000 most variable regions of 376 DNAse I hypersensitivity profiles from the ENCODE project, including cell lines, primary tissue, and embryonic tissue. Clusters were identified by a Pearson's correlation-based clustering method, see materials and methods. **(B-C)** Ranked plots of overlap analysis between cluster specific peak sets generated from (A) and lower (B) and higher (C) accessible region sets from Ctrl.Ctrl(A) vs P8$_1$Ctrl(A). One-tailed hypergeometric test. **(D)** Heatmaps showing normalized ATAC-seq signal +/- 2kb centered on peak summits for the ENCODE adult

IPS/progenitor cluster region set. **(E-F)** Two example genomic loci showing a gain in ATAC-seq signal at ENCODE IPS/progenitor cluster specific peaks.

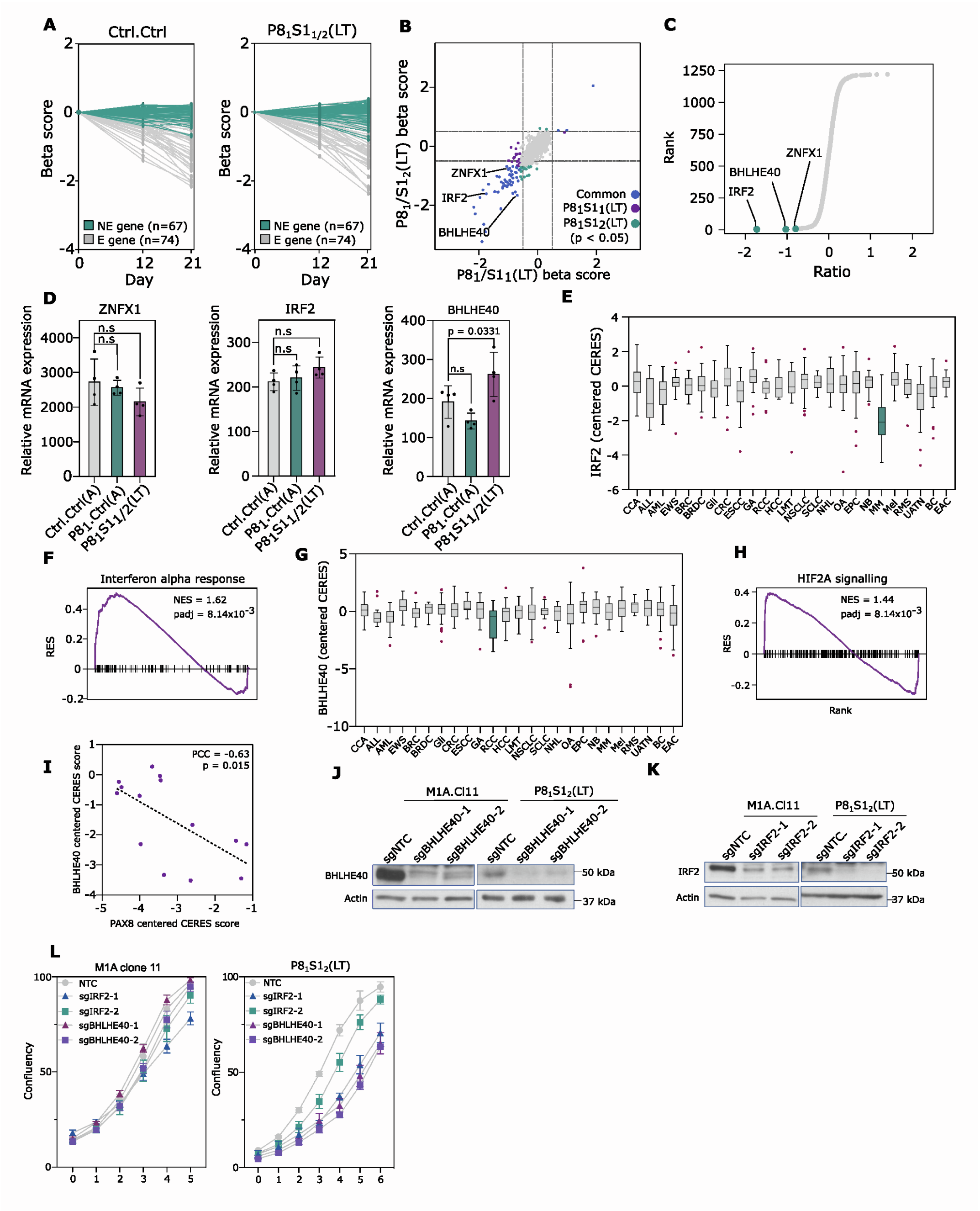

Figure S6. Gene dependencies in lineage factor inhibition resistant ccRCC cells, related to Figure 5. (A) Beta scores of essential and non-essential genes throughout the screen for the control arm (left) and experimental arm (right). **(B)** Beta scores for the two experimental arms of the screen, P8$_1$S1$_1$(LT) versus P8$_1$S1$_2$(LT). Beta scores were calculated using the fold change of the top three depleted sgRNAs per gene relative to the plasmid library, from two replicates. *P*-value calculated by permutation method using MAGeCK. **(C)** Ranked log$_2$ ratio of P8$_1$S1$_{1/2}$ / Ctrl.Ctrl beta scores for

day 21 of the screen. **(D)** Fold change in mRNA expression versus Ctr.Ctrl cells from RNAseq expression data. Adjusted *p*-values calculated using DEseq. **(E)** Genetic dependency data from the DepMap project. IRF2 centered CERES dependency scores across 25 lineages, with ≥ 10 cell lines per lineage. **(F)** GSEA plot of the interferon alpha response signature from the hallmarks collection, for $P8_1$Ctrl(A) vs $P8_1S1_{1/2}$(LT). **(G)** Genetic dependency data from the DepMap project. BHLHE40 centred CERES dependency scores across 25 lineages, with ≥ 10 cell lines per lineage. **(H)** GSEA plot of a HIF2A target gene signature for $P8_1$Ctrl(A) vs $P8_1S1_{1/2}$(LT). See materials and methods for signature derivation. **(I)** Correlation of BHLHE40 and PAX8 centered CERES scores for PAX8 sensitive and SMARCB1 mutant *VHL*-mutant ccRCC cell lines. Pearson's correlation coefficient. **(J-K)** Western blot of BHLHE40 and IRF2 expression in the indicated cell lines. **(L)** Confluency-based cellular proliferation assay using the cell lines from panels (J-K). Three technical replicates per condition. Error bars are SD. Kruskal-Wallis test. CCA: cholangiocarcinoma, ALL: acute lymphoblastic leukemia, AML: acute myeloid leukemia, EWS: Ewing sarcoma, BRC: breast carcinoma, BRDC: breast ductal carcinoma, Gli: Glioma, CRC: colorectal adenocarcinoma, ESCC: esophageal squamous cell carcinoma, GA: gastric adenocarcinoma, RCC: renal cell carcinoma, HCC: hepatocellular carcinoma, LMT: lung mesothelioma, NSCLC: non-small cell lung cancer, SCLC: small cell lung cancer, NHL: non-Hodgkin lymphoma, OA: ovarian adenocarcinoma, EPC: exocrine pancreatic cancer, NB: neuroblastoma, MM: multiple myeloma, Mel: melanoma, RMS: rhabdomyosarcoma, UATN: upper aerodigestive tract neoplasm, BC: bladder carcinoma, EAC: endometrial adenocarcinoma.

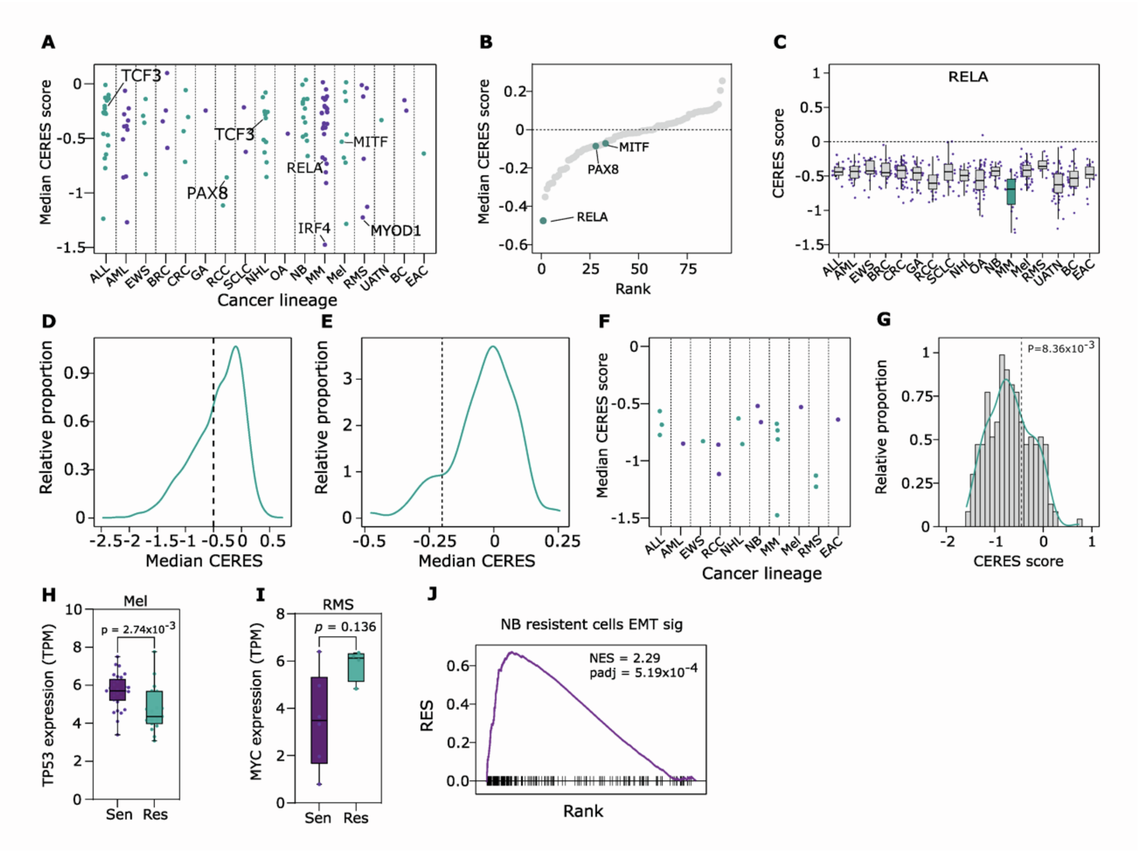

Figure S7. Lineage factor independence across cancer cell lines, related to Figure 6. (A) Dot plot of the median CERES score across cell lines in a particular lineage, for putative lineage specific TF dependencies after filtering (LDscore ≤ -1.2, P<0.05). **(B)** A ranked plot of the median CERES scores across all cell lines (across all lineages) for TFs identified as lineage specific. **(C)** Box plot of CERES scores for the gene RELA across all lineages with identified specific dependencies (n=17). Highlighted lineage, MM, is the lineage for which RELA was identified as a specific dependency. **(D)** Frequency distribution of the median CERES scores for each identified lineage dependency from panel (A) across all cell lines in their respective lineage. **(E)** Frequency distribution of the median CERES scores for each identified lineage dependency from panel (A) across all cell lines. **(F)** Dot plot of the median CERES score across cell lines in a particular lineage, for prospective lineage specific TF dependencies after three successive rounds of filtering (1) LDscore ≤ -1.2, P<0.05,

(2) ≥ 50% of cell lines with the lineage have a CERES score of ≤ -0.5 for a prospective LD and (3) the median CERES score of LD across all cell lines ≥ -0.2. **(G)** Frequency distribution of the CERES score for each LD from panel (A), in each cell line of their respective lineage. For lineages which contain >1 LD, an average is taken across LDs in each cell line. Distribution statistically deviated from normal determined using the Shapiro Test. **(H)** Box plot of normalized TP53 gene expression (transcripts per million -TPM) in lineage sensitive (n=30) and resistant (n=19) cell lines from the melanoma lineage. Kruskal-Wallis test. For boxplots, center line shows the median, the box bounds represent the first and third quartiles and the whiskers extend to the highest and lowest values. **(I)** Box plot of normalized MYC gene expression (TPM) in lineage sensitive (n=6) and resistant (n=4) cell lines from the rhabdomyosarcoma lineage. Kruskal-Wallace test. For boxplots, center line shows the median, the box bounds represent the first and third quartiles and the whiskers extend to the highest and lowest values. **(J)** GSEA plot of the hallmarks EMT signature in lineage sensitive vs lineage resistant neuroblastoma cell lines.

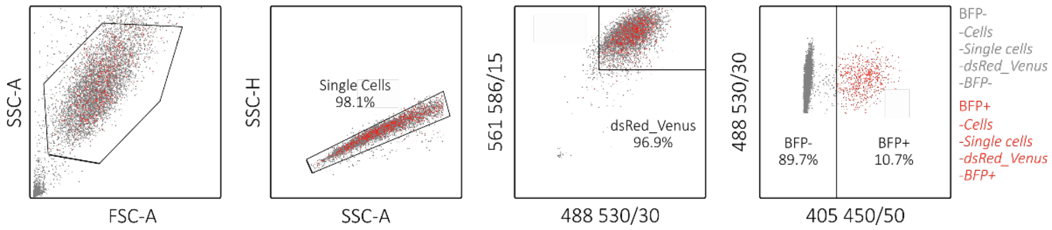

Figure S8, FACS gating strategy, related to Figure 1 and Figure S2.
